# The Temporal Signature of Memories: Identification of a General Mechanism for Dynamic Memory Replay in Humans

**DOI:** 10.1371/journal.pbio.1002528

**Published:** 2016-08-05

**Authors:** Sebastian Michelmann, Howard Bowman, Simon Hanslmayr

**Affiliations:** 1 School of Psychology, University of Birmingham, Birmingham, United Kingdom; 2 School of Computing, University of Kent, Canterbury, United Kingdom; Radboud University Nijmegen, NETHERLANDS

## Abstract

Reinstatement of dynamic memories requires the replay of neural patterns that unfold over time in a similar manner as during perception. However, little is known about the mechanisms that guide such a temporally structured replay in humans, because previous studies used either unsuitable methods or paradigms to address this question. Here, we overcome these limitations by developing a new analysis method to detect the replay of temporal patterns in a paradigm that requires participants to mentally replay short sound or video clips. We show that memory reinstatement is accompanied by a decrease of low-frequency (8 Hz) power, which carries a temporal phase signature of the replayed stimulus. These replay effects were evident in the visual as well as in the auditory domain and were localized to sensory-specific regions. These results suggest low-frequency phase to be a domain-general mechanism that orchestrates dynamic memory replay in humans.

## Abstract 2

Study of the mental replay of audio and video clips shows that the phase of an 8-Hz oscillation carries a temporal code of individual visual and auditory memories.

## Author Summary

A remarkable ability of the human brain is that it can mentally replay past episodes. For instance, if one remembers the last movie one has seen, one can vividly evoke parts of this event in a temporally highly structured manner. This implicates a neural mechanism that temporally guides the brain through memory retrieval as a sensory trace unfolds over time. However, those mechanisms are weakly understood, because little is known about the temporal dynamics of memory replay. Using a new analysis method, we detect the replay of temporal patterns in a memory task that requires participants to mentally replay short sound or video clips. We show that the phase of a low-frequency oscillation carries a temporal signature that is unique to specific stimulus content (either visual or auditory) and signifies its replay in memory. Importantly, the replay of temporal signatures is localized in processing regions specific for each sensory modality and is related to decreases in low-frequency power in the same regions. Our study provides first insight that our brain codes information of dynamic stimuli in the phase of low-frequency neural time series and that these time series can be replayed purely from episodic memory.

## Introduction

Episodic memories are dynamic, multisensory events that are coded in our memory system. If you remember the last time you had dinner at your favorite restaurant, you will probably recall the person you were with, the music playing in the background, and the smell and taste of that delicious food. Whenever we re-experience episodic memories this way, the events unravel in front of our mind in a temporal order. Even subparts of these episodes, such as the movement of lips in a conversation or parts of the background melody, have an inherent temporal dynamic to them. Given this abundance of temporal structure in our memories, it is rather surprising how limited our understanding is as to how human brains orchestrate such dynamic memory replay. Here, we address this question for the first time, to our knowledge, and identify a neural mechanism that carries the temporal signature of individual dynamic episodic memories. By cuing dynamic memories of auditory and visual content, we were able to detect the presence of phase patterns in the electroencephalographic (EEG) signal that indicate the replay of individual auditory or visual stimuli in memory. Temporal signatures were carried by a frequency that was markedly similar in two sensory domains (~8 Hz), they appeared in sensory-specific regions, and they were related to decreases in power in the same frequency.

Previous findings suggest that perception is not continuous but, instead, is rhythmically sampled in discrete snapshots guided by the phase of low alpha (~7–8 Hz) [1–3], which suggests a pivotal role of low alpha phase for providing a temporal structure during perception [4–8]. Accordingly, recent studies showed that low-frequency phase carries reliable information about stimulus content [9,10]. This key role of oscillatory phase during perception makes it a prime candidate to also organize the replay of neural representations in episodic memory, which is an untested prediction to date.

A ubiquitous electrophysiological signature of successful memory processing is a pronounced power decrease in low frequencies, especially in alpha [11–15]. On a theoretical level, decreases in alpha power affect neural processing in two ways. First, they promote increased neural activity, as reflected by increased neural firing rates and increased blood-oxygen-level dependent (BOLD) signal [16–18]. Importantly, even when alpha power is decreased, its phase still rhythmically modulates firing rates [7]. Second, alpha power decreases reflect a relative de-correlation of neural activity and thereby index an increase in information coding capacity [8]. Accordingly, a mechanism by which decreases in alpha power allow for the temporal organization of information via phase has been proposed in perception [7]; however, whether memory replay is guided by a similar mechanism is an open question [8].

The reinstatement of neural patterns in memory can be detected with multivariate analysis methods such as representational similarity analysis (RSA) [19]. This approach has been successfully applied in functional magnetic resonance imaging (fMRI) [20,21], EEG/MEG [9,22–26] and intracranial EEG (iEEG) [27,28]. However, even though some previous studies were able to decode information from oscillatory patterns, the mechanism by which oscillations carry mnemonic information remains completely unclear. This is because most prior studies either settle for classification of reactivated memories and thus do not aim for mechanistic explanations of memory replay or because they use static stimuli and analysis procedures.

**Fig 1:**
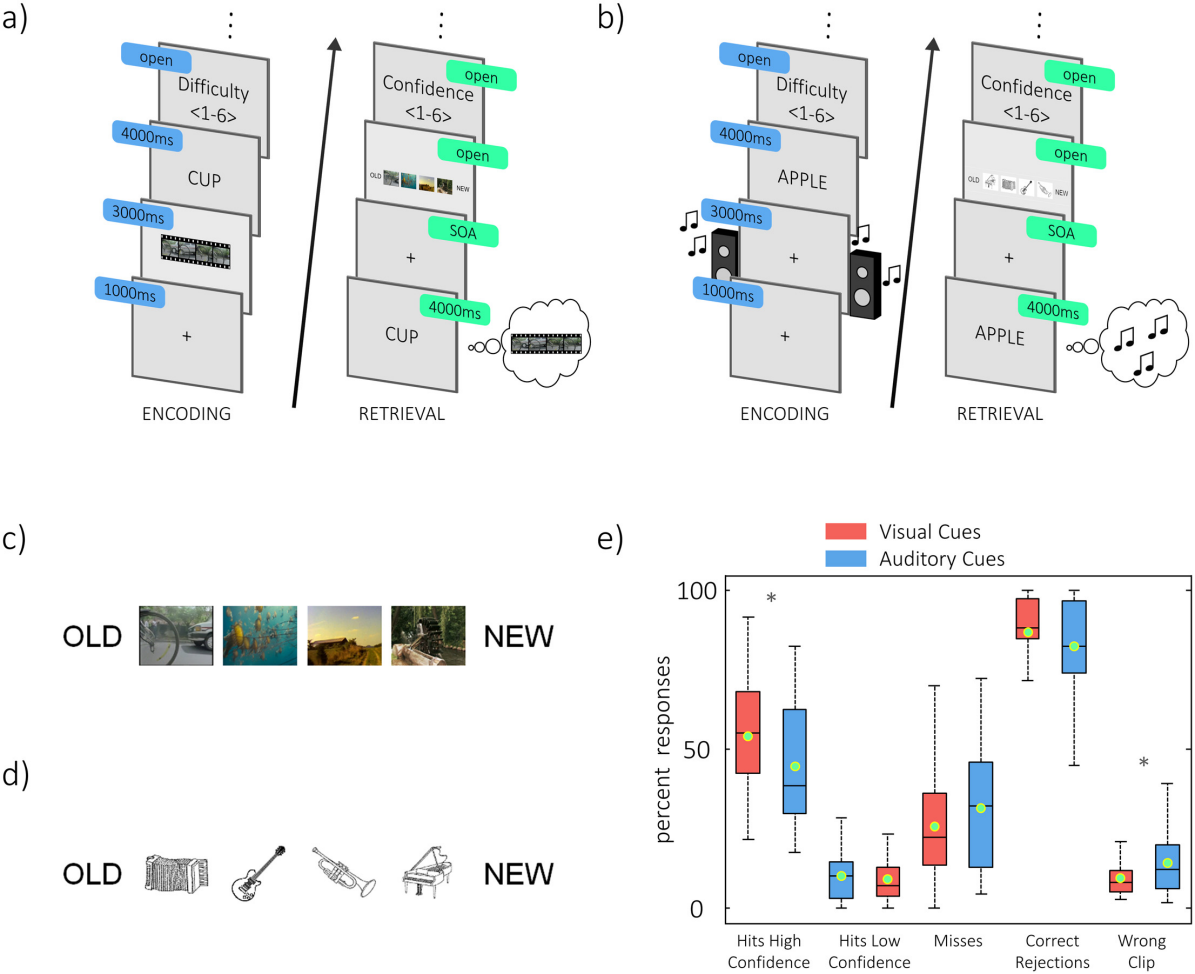
Experimental design and behavioral results. (a,b) Trial sequences are shown for the visual (a) and the auditory session (b). In the encoding block (a,b left), participants were presented with a dynamic stimulus that played for 3 s and was immediately followed by a word cue, which was presented for 4 s. During encoding, subjects learned 120 associations between four repeatedly shown dynamic stimuli and 120 different words. At the end of every encoding trial, the perceived difficulty of the association was rated on a scale from 1 to 6. In the retrieval block (a,b right), subjects only saw the static word cue and were asked to vividly recall the dynamic stimulus it was associated with. Cues from encoding were mixed with 60 new words that served as distractors. Note that during encoding, the word cue was shown after the dynamic stimulus, avoiding sensory overlap between encoding and retrieval. Subjects were then asked to indicate the stimulus they recalled. Response options (c,d) consisted of four small screenshots of the video clips in the visual condition (c) and of four small instruments, representing the sounds, in the auditory condition (d). Additionally, the response option “NEW” was presented to indicate that the word was not presented in the encoding block (distractor item), the response option “OLD” was available to indicate that subjects remembered learning the word, but could not recall the content it was associated with. After responding, subjects were asked to rate the confidence in their answer on a scale from 1 to 6 (a,b right). (e) Behavioral performance is plotted. Hits are trials in which the correct associate was remembered (i.e., video or sound). A rating of high confidence was considered a rating > 4. Misses were defined as those trials in which the associate was not remembered and as those trials in which a word cue was wrongly named as a distractor. Boxes are 25th and 75th percentiles around the median, whiskers represent minimum and maximum, green points are arithmetic means. * *p* < 0.05.

We overcome these central limitations by testing whether a temporal signature that is present while a video or a sound clip is perceived is actively reproduced by the brain during retrieval. By temporal signature we mean a sequence of electrophysiological activity that is specific to an individual stimulus. To this end, we test the mechanistic hypothesis that low-frequency power decreases are linked with the reinstatement of such stimulus-specific phase patterns. A paradigm was used in which memories of dynamic content are cued by a static word (see Fig 1a–1d). In a visual and in an auditory condition, we asked subjects to watch (or listen to) 3-second-long video or sound clips and then to associate the respective stimulus with a word. Importantly, only four videos/sounds were repeatedly associated with different words. In the retrieval block, we then only presented the word cue (or a distractor word) under the instruction to vividly replay the associated video or sound. Note that there was no overlap in sensory input between the video/sound and the word, enabling us to investigate purely memory-driven reinstatement of temporal signatures.

**Fig 2:**
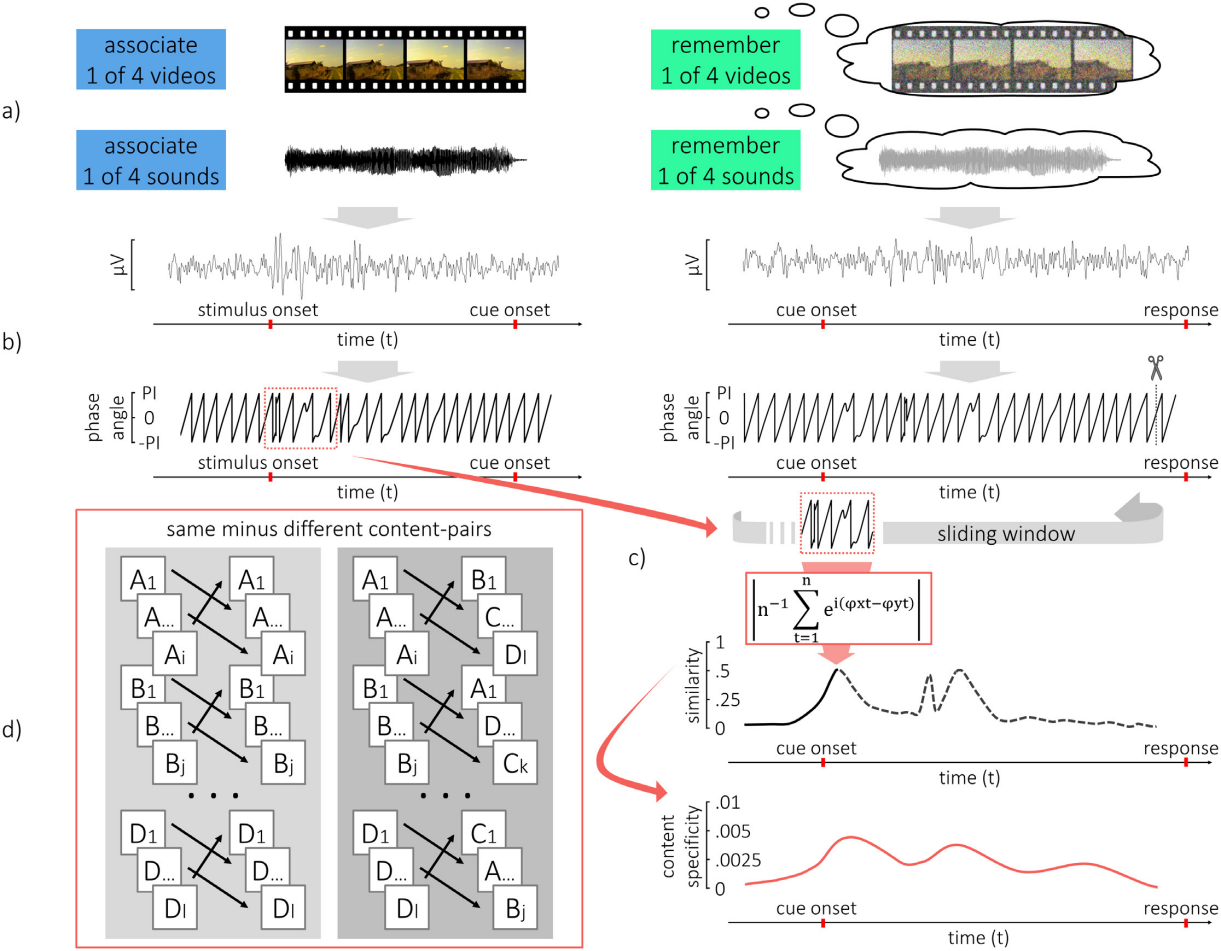
Detection of content-specific reinstatement of temporal patterns. (a) During encoding, subjects associated one of four videos with a different cue word in every trial in the visual condition, or they associated one of four sounds with different cue words in the auditory condition (a, left). During retrieval, subjects only saw the static word cue on the screen and were asked to recall the corresponding dynamic stimulus (a, right). (b) At every electrode, the oscillatory phase for a frequency of interest was extracted from the EEG activity. A time window from encoding was then selected and the time course of phase in this window was compared to retrieval. (c) A sliding window was used to assess the similarity, based on the constancy of phase angle differences over time (Single Trial Phase Locking Value [32,33]). This measure made it possible to assess similarity between single trials in which the strength of similarity ranged from 0 to 1, even though an oscillatory pattern was compared. Therefore similarity could be averaged across trials, time and participants. To avoid confounds from the response and the response scale, the data was cut at the end of the retrieval trial and the window was slid out, back into the pre-stimulus interval. This was done for trial-pairs of same content (e.g., learning A, remembering A) and for trial-pairs of different content (e.g., learning A, remembering D). (c,d) The difference in similarity between pairs of same and pairs of different content was interpreted as evidence for content specific reinstatement of temporal patterns.

We hypothesize that we will find content-specific temporal signatures in those frequency bands that show pronounced power decreases during episodic memory retrieval [8]. Applying the logic of RSA to measures of phase-based similarity, we designed a new method that can detect content-specific signatures in neural time series in which the exact onset of replay is not known (see Fig 2), which is the case in our retrieval phase. To our knowledge, this is the first time that a method can use oscillatory phase patterns to decode content from activity that is not time-locked. We assess reinstatement in the auditory and in the visual modality in order to validate our novel, dynamic RSA method and to test for a domain-general memory replay mechanism. Whole-brain activity was measured via high-density EEG, and individual MRIs were collected to increase the fidelity of source localization.

## Results

### Behavioral Performance

Behavioral results are shown in Fig 1e. In the visual session, participants remembered on average 53.92% (standard deviation [s.d.] = 17.56%) of the video clips with high confidence (rating > 4), and they further remembered 9.97% (s.d. = 7.62%) of the clips with low confidence. However, in order to increase the signal-to-noise ratio, hits with a low confidence rating were not included in further analysis. In the auditory session, 44.44% (s.d. = 19.8%) of the audio clips were remembered with high confidence, which was significantly less than in the visual condition (*t*_23_ = -2.81, *p* = 0.01). An additional 9.06% (s.d. = 6.9%) of the audio clips were remembered with low confidence. In accordance, the number of misses tended to be lower in the visual session (mean 25.66%, s.d. = 17.56%) than in the auditory session (31.46%, s.d. = 19.15%, *t*_23_ = -1.91, *p* = 0.07). Another trend was observed toward a better identification of distractor words in the visual session (*t*_23_ = 1.92, *p* = 0.07), in which 86.88% (s.d. = 13.03%) of the distractors were correctly rejected, while subjects correctly identified only 82.43% (s.d. = 15.86%) of the distractors as new words in the auditory session. Keeping with the slightly better performance for visual compared to auditory memories, the wrong video clip was less frequently selected in the visual (9.4%, s.d = 6.33%) condition, compared to the wrong sound clip in the auditory session (14%, s.d. = 9.69%, *t*_23_ = -2.86, *p* < 0.01).

### Successful Memory Is Associated with Low-Frequency Power Decreases

**Fig 3:**
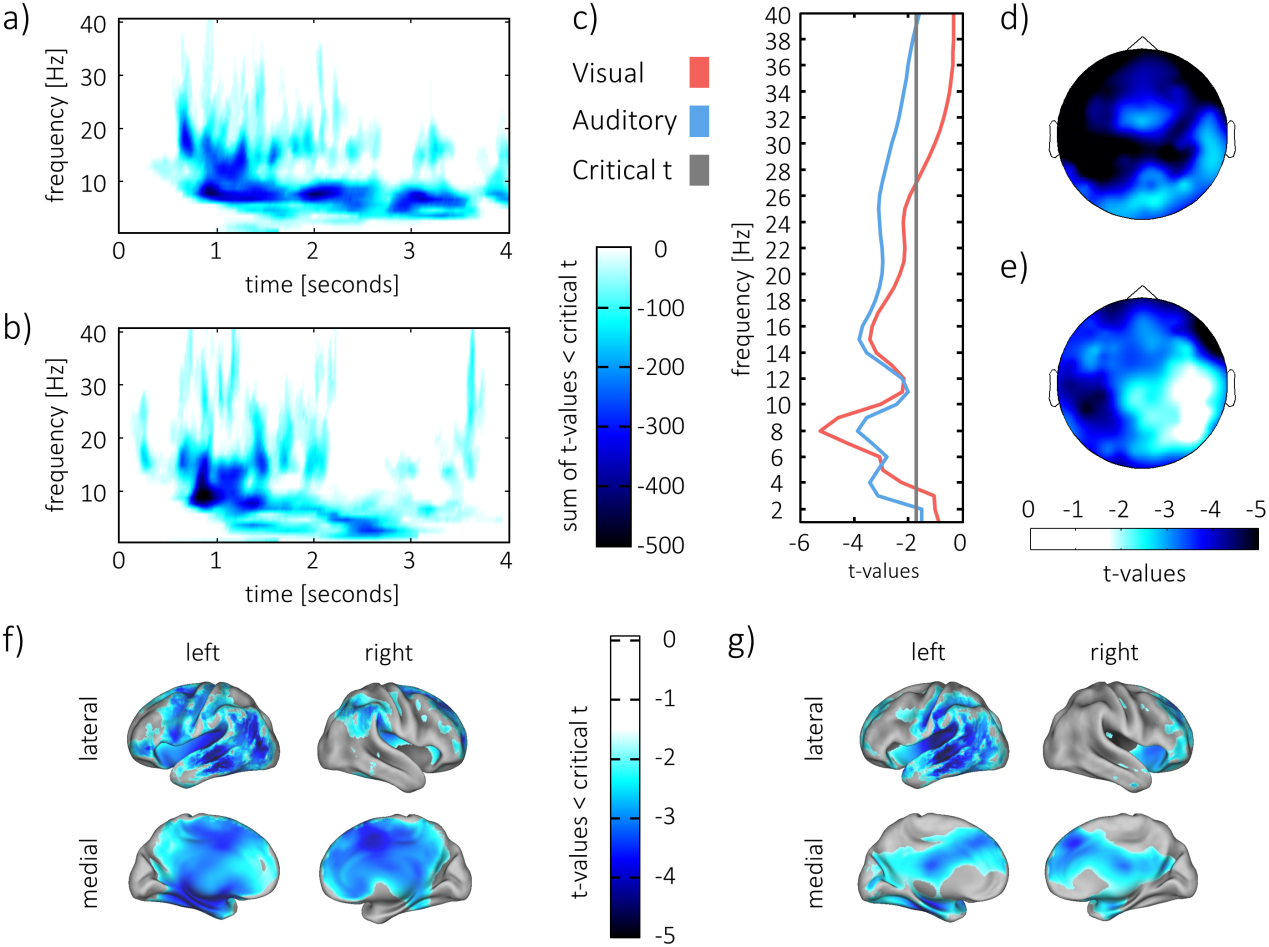
Contrast of hits and correct rejections. Successful memory reinstatement was associated with a cluster of strong power decreases in the lower frequencies (<30 Hz). (a,b). The sum of t-values across all electrodes in the cluster of significant differences is plotted in the visual condition (a) and in the auditory condition (b). (c) The t-statistic of power decreases that were averaged over electrodes and time, showing a peak at 8 Hz. (d,e) Topography of power decreases in the visual condition (d) and in the auditory condition (e). Power decreases are plotted as t-values of average difference at 8 Hz between 0 and 4 s during retrieval. (f,g) Reconstruction of 8 Hz power difference in source space using an “lcmv” beamforming-algorithm in the visual (f) and in the auditory (g) condition.

In order to identify oscillatory correlates of memory reinstatement, trials in which subjects were presented with a memory cue and strongly reinstated the content (i.e., high confidence hits) were contrasted with trials in which participants were presented with a distractor item and correctly indicated it as a new item (i.e., correct rejections). As expected, successful memory retrieval was associated with strong power decreases in the low frequencies (<30 Hz); power increases did not survive statistical testing, including the gamma frequency range (up to 140 Hz). The clusters that survived multiple comparisons correction (see Materials and Methods) are shown in Fig 3. Stronger power decreases for hits were obtained when compared to correct rejections in the visual (Fig 3a, *p* < 0.001) and in the auditory condition (Fig 3b; *p* < 0.001).

The same results emerged when a contrast was built between high confidence hits and trials in which subjects failed to remember the corresponding video or sound clip; that is, when they either failed to retrieve the correct associate or judged an old item as new (see contrast of hits and misses, S1 Text, S1 Fig). This further emphasizes the link of power decreases to successful memory reinstatement.

To identify the frequencies that showed the strongest decrease in oscillatory power, the power difference across all electrodes and time points in the retrieval interval was averaged and subjected to a *t*-test. Power decreases peaked at 8 Hz (see Fig 3c) when contrasting hits and correct rejections in the visual condition (*t*_23_ = -5.2696, *p* < 0.001) and in the auditory condition (*t*_23_ = -3.86, *p* < 0.001).

In the visual condition, these 8-Hz power decreases displayed a broad topography that showed a parietal maximum over the left hemisphere and frontal maxima over both hemispheres (Fig 3d). In the auditory condition, power decreases at 8 Hz were equally broad. Maxima were located over left parietal and right frontal regions (Fig 3e).

In order to identify brain regions in which power decreases were maximal at 8 Hz, sources of the difference between hits and correct rejections were reconstructed for that frequency (see Materials and Methods). Statistical testing was run unrestricted on the whole brain level. After multiple comparison correction (see Materials and Methods), a cluster of significant differences emerged in the visual (*p* < 0.001) and in the auditory (*p* = 0.002) condition. Clusters of power decreases were broad and did not show statistical differences between the visual and the auditory condition. In the visual condition (Fig 3f), the cluster of significant differences spanned parietal, temporal, and frontal regions of the left hemisphere and mid-frontal and parietal regions of the right hemisphere. In the auditory condition (Fig 3g), power decreases spanned left parietal, temporal, mid-frontal, and right frontal regions.

### Temporal Patterns Differentiate between Content during Encoding

An important requirement for the detection of replay of temporal patterns during memory retrieval is that the stimulus content itself elicits a distinct time course of activity in the first place, i.e., while being perceived during encoding. In order to test this prerequisite, a modified version of the pairwise phase consistency (PPC) [29] was contrasted between pairs of trials in which the same content was presented and pairs of trials that were of different content. This method assesses the degree of phase similarity that is specifically shared by trials that are instances of the same stimulus (i.e., content specificity of phase).

**Fig 4:**
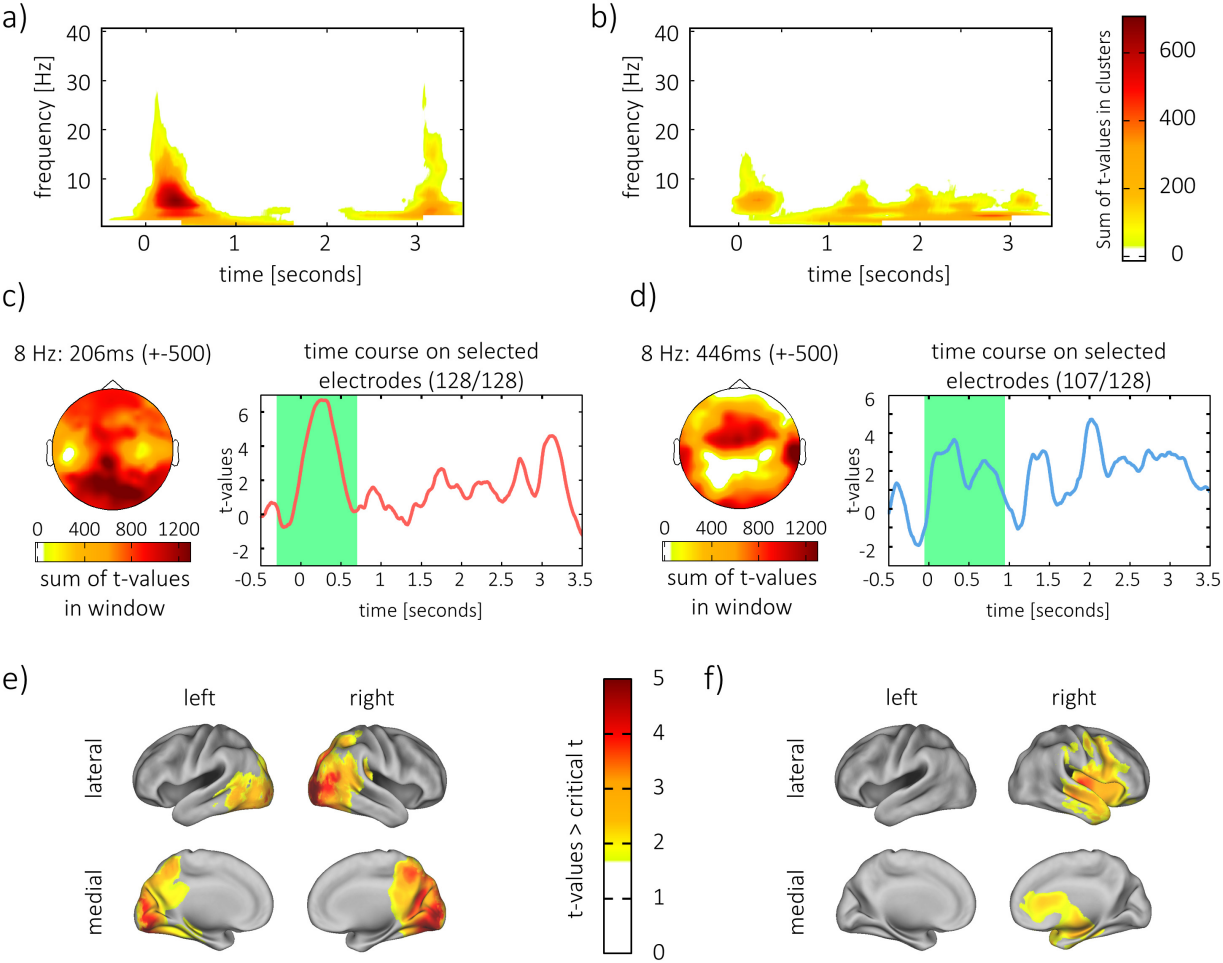
Content specificity of phase during encoding. Broad clusters of difference in phase similarity across time. Frequency and electrodes were observed (a) in the visual condition and (b) in the auditory condition. t-values were summed across electrodes in the cluster to display the results. (c,d) Topography of 8 Hz content specificity and time course of differentiation (c) in the visual condition and (d) in the auditory condition. The time course (c,d right) is the t-statistic of averaged content specificity, across all electrodes that are included in the strongest cluster. The green window marks the time window of 1 s around the center of the strongest cluster. (e) Source reconstruction of content specificity in the visual condition and (f) in the auditory condition. For consistency, sources were tested and plotted on the averaged similarity across the encoding time window of 1 s around the peak of the strongest cluster (green window). Likewise, topographies (c,d left) show summed t-values on the scalp of content specificity across this time window.

Content specificity of phase was assessed for every frequency band between 1 and 40 Hz. The time window for statistical testing was chosen between 500 ms pre-stimulus and 3500 ms after stimulus onset, to account for the temporal smearing of the wavelet decomposition. Importantly, the combination of trials was carefully balanced to avoid any possible bias (see Materials and Methods). After correction for multiple comparisons, significant differences were obtained in both conditions in the form of two broad clusters in the visual (*p* < 0.001, *p* = 0.003, Fig 4a) and one broad cluster in the auditory condition (*p* < 0.001, Fig 4b). Importantly, the clusters included 8 Hz, which showed the strongest memory effects during replay (see above).

We hypothesized that we would later find reappearing temporal patterns in the frequency band of 8 Hz during retrieval; furthermore, content specificity during encoding is a requirement for the detection of these patterns. For these reasons, temporospatial clusters in the data were now identified in which the 8 Hz time course was maximally content specific. Hence, the statistical analysis was now restricted to 8 Hz only. After multiple comparison correction, the cluster at encoding in which content could most reliably be differentiated (i.e., the cluster with the lowest *p*-value) was selected for further analysis.

In the visual condition, this cluster was identified between -152 ms and 564 ms (*p* < 0.001). Note that post-stimulus effects are temporally smeared into the pre-stimulus interval due to wavelet filtering. One further cluster was observed between 2,650 ms and 3,300 ms (*p* = 0.016).

In the auditory condition, the most reliable cluster of content specificity was identified in a time window between 22 ms and 871 ms (*p* = 0.002). Two further clusters were observed ranging from 1,818 ms to 2,627 ms (*p* = 0.003) and from 1,203 ms to 1,504 ms (*p* = 0.047). Therefore, in both domains, early and later time windows distinguished between different stimuli, reflecting the dynamic nature of the stimulus material.

A 1 s time window was then defined around the center of the most content-specific cluster. In the visual condition, this center was located at 206 ms; thus, the window ranged from -294 ms to 706 ms (Fig 4c, right). Differences in phase similarity between trials of same and different content showed a clear visual topography within this window, i.e., the highest t-values were observed over posterior regions of the scalp (Fig 4c, left).

In the auditory condition, the 1 s window was centered at 446 ms (Fig 4d, right), ranging from -54 ms to 946 ms. The topography of differences within that window showed a typical auditory distribution (Fig 4d, left), i.e., high t-values were observed at fronto-central electrodes [30].

Sources of the average difference in phase similarity between same and different content combinations were reconstructed for the 1 s windows to identify the origin of content specificity in that window. *t*-tests were corrected for multiple comparisons on the whole-brain level. In the visual condition, a cluster of significant difference (*p* < 0.001) emerged in visual regions of the cortex, covering the occipital lobe as well as parts of the parietal lobe (Fig 4e). The cluster exhibited a peak in the right middle occipital gyrus (MNI: 10; -100; 10; BA: 18). In the auditory condition, differences in similarity (*p* = 0.004) were lateralized to the right hemisphere, which is in line with studies finding lateralization of musical processing to this hemisphere [31]. Differences covered temporal and frontal areas, including primary and secondary auditory processing regions (Fig 4f). The auditory cluster peaked in right sub-lobar insula (MNI: 40; -20; 0).

### Temporal Patterns Indicate Replay of Visual and Auditory Content

The crucial quest to identify a replay of temporal patterns from encoding during retrieval is challenged by the non-time-locked nature of retrieval. Indeed, replay of memory content during retrieval could happen at any point after presentation of the retrieval cue, with the exact onset varying from trial to trial. Moreover, we assumed that any temporal pattern from encoding could be replayed at any time during retrieval.

We therefore developed a procedure that is not affected by these time shifts; specifically, we assessed the similarity between encoding and retrieval with a sliding window approach. To this end, phase similarity between combinations of encoding and retrieval time windows was computed using a variation of the single-trial phase locking value (S-PLV) [32,33], namely the similarity of phase angle differences over time (see Materials and Methods). This method is less susceptible to noise and allows for an estimation of similarity between two time windows in non-time-locked data. Again, phase-similarity of encoding-retrieval pairs that were of the same content (e.g., perceiving A, remembering A) was contrasted with the similarity of pairs that were of different content (e.g., perceiving B, remembering D).

The time window that contained the temporal pattern from encoding was selected based on the highest content specificity of phase during encoding (see above). The width of the window amounted to 1 s (8 cycles) around the center of the cluster that was located at 206 ms (-294 to 706 ms, see Fig 4c, right) in the visual condition and at 446 ms in the auditory condition (-54 to 946 ms, see Fig 4d, right). Since activity at encoding, under the null-hypothesis, would be independent from activity at retrieval, the specific selection of a time window, based on results from encoding, can be used to increase the signal to noise ratio, without risking circular inference.

Phase-similarity to this predefined encoding window was now assessed by sliding the window over the whole retrieval episode. To slide the window into retrieval, the pre-stimulus interval between -500ms and 0ms was used as padding. To slide it out at the end of the trial, the pre-stimulus interval between -1,000 ms and -500 ms was used as padding (this was done because later time points were unsuitable for padding due to contamination with similar perception and responses). Note that the similarity at time point 0 is then assessed by comparing the encoding window to the retrieval window between -500 ms and 500 ms, and similarity at 4 s is assessed by comparing the encoding window to the concatenated retrieval window of 3,500 ms to 4,000 ms and -1,000 ms to -500 ms.

**Fig 5:**
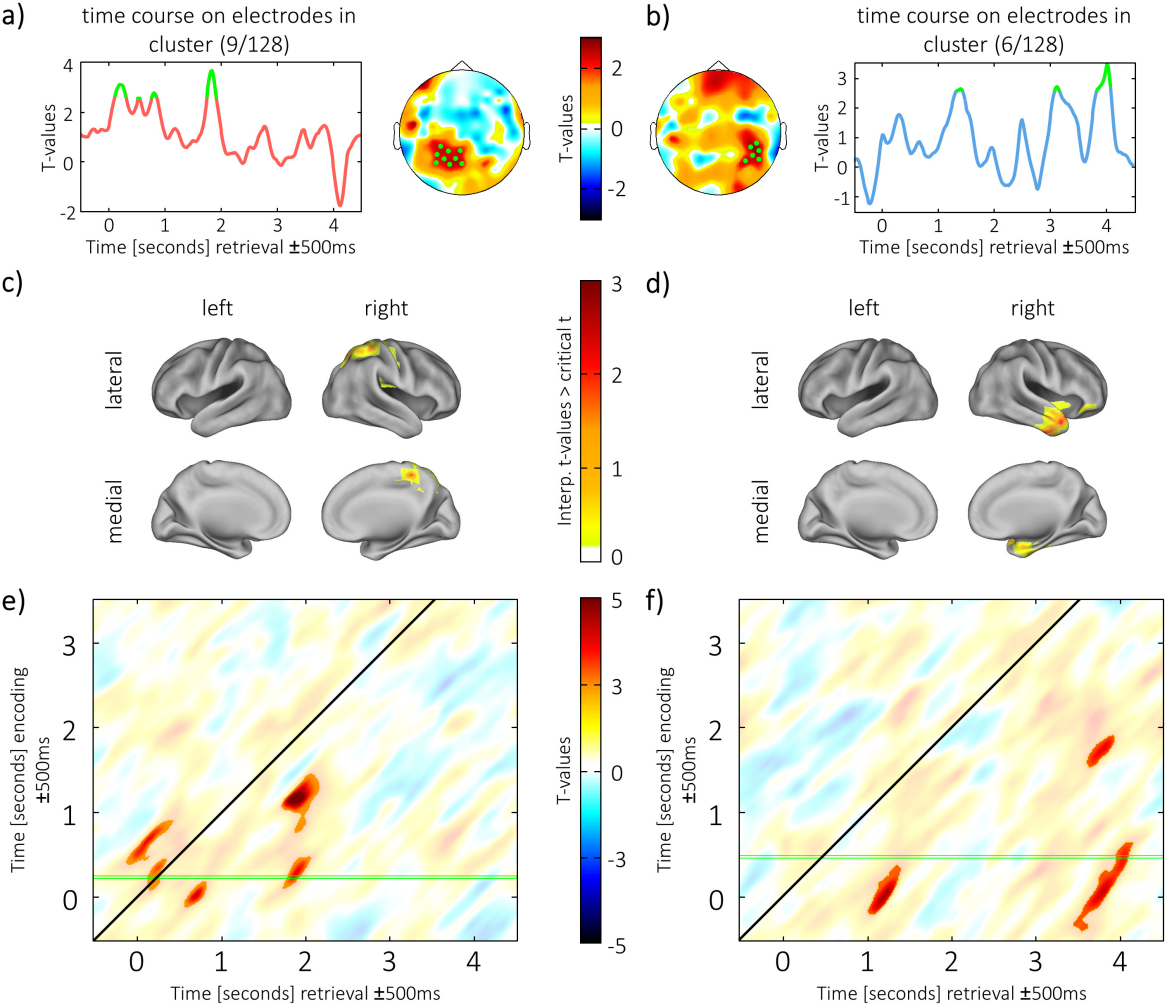
Encoding-retrieval similarity. (a) Topography of visual cluster and time course on electrodes in the cluster. (b) Topography of auditory cluster and time course on electrodes in the cluster. Electrodes in the cluster and time points that exceed threshold are highlighted in green. (c,d) Source reconstruction of encoding-retrieval similarity between 0 and 4 s of retrieval in the visual condition (c) and in the auditory condition (d). Statistical testing was run unrestricted on the whole brain level, and for each condition the maximal cluster (i.e., with the highest summed *t*-values) was plotted. (e,f) Encoding-retrieval similarity in the cluster (from a,b) between every time point of encoding and every time point of retrieval in the visual condition (e) and in the auditory condition (f). Clusters of significant differences are unmasked; remaining data is masked with transparency. The temporal imprecision of ±500 ms is due to the width of the sliding window. The green lines in e and f highlight the original time window at encoding that is displayed in (a) and (b) (for e,f).

The phase-similarity of the encoding window to episodes of replay of the same video/sound was now contrasted with the phase-similarity to episodes of replay where a different content was replayed from memory. The *t*-statistic was computed for every electrode on the averaged difference between same versus different combinations between 0 and 4 s. A cluster-based permutation test indicated replay of encoding phase patterns during retrieval for both the visual (*p* = 0.002) and the auditory condition (*p* = 0.01). In the visual replay condition, the cluster of significant differences emerged over left parietal regions (Fig 5a, right). In the auditory replay condition, a cluster of significant differences was observed over right posterior temporal areas (Fig 5b, left). This signifies strong evidence for mnemonic replay of temporal patterns.

To test for frequency specificity, the same analysis was performed for 5 Hz and 13 Hz, which are approximately in a golden ratio relationship to 8 Hz (i.e., maximally different in phase) [34]. Two further control frequencies were tested that showed peaks in power decreases in at least one of the conditions, namely 4 Hz and 15 Hz. To this end, time windows from encoding were selected with the same criteria as for 8 Hz; electrodes for testing were again restricted to the electrodes in the significant cluster from encoding. Furthermore, the time window was likewise built from 8 cycles of the corresponding frequency. However, no effects were found in the visual or in the auditory condition for any of the control frequencies, suggesting that temporal reinstatement of phase patterns was specific to 8 Hz.

The temporal profile of the replay effect was then inspected by averaging phase similarity across electrodes within the cluster of significant differences. A *t*-test was computed at every time point, applying a probability of error below 0.01. For visual material, four episodes of replay could be identified (Fig 5a, left), in which a one-sided test exceeded the critical threshold (*t*_23_ = 2.5). These episodes peaked at 203 ms (*t*_23_ = 3.09, *p* = 0.003), 547 ms (*t*_23_ = 2.51, *p* = 0.01), 828 ms (*t*_23_ = 2.75, *p* = 0.006), and 1,844 ms (t_23_ = 3.65, *p* < 0.001). For the auditory material, three episodes exceeded the critical *t*-value (Fig 5b, right), peaking at 1,406 ms (*t*_23_ = 2.64, *p* = 0.007), 3,125 ms (*t*_23_ = 2.7, *p* = 0.006), and 4,016 ms (*t*_23_ = 3.47, *p* = 0.001).

To reveal whether the encoding-retrieval similarity effects were maximal in material-specific (i.e., visual/auditory) brain regions, encoding-retrieval similarity was assessed on the source level. Statistical testing was run unrestricted on the whole brain level, and for each condition the maximal cluster (i.e., with the highest summed *t*-values) was plotted. For the visual material, the strongest cluster of encoding-retrieval similarity showed a peak in the superior parietal lobule (MNI: 20; -50; 60, BA: 7, see Fig 5c), overlapping with the similarity effects during encoding (compare to Fig 4e) and in line with studies finding parietal lobe contributions to episodic memory retrieval [35,36]. For the auditory material, similarity effects showed a peak in the right inferior temporal gyrus (MNI: 50; -10; -44, BA: 20, see Fig 5d) also overlapping with the similarity effects during encoding (compare Fig 4f) and in line with previously reported effects on memory for music [37].

**Fig 6:**
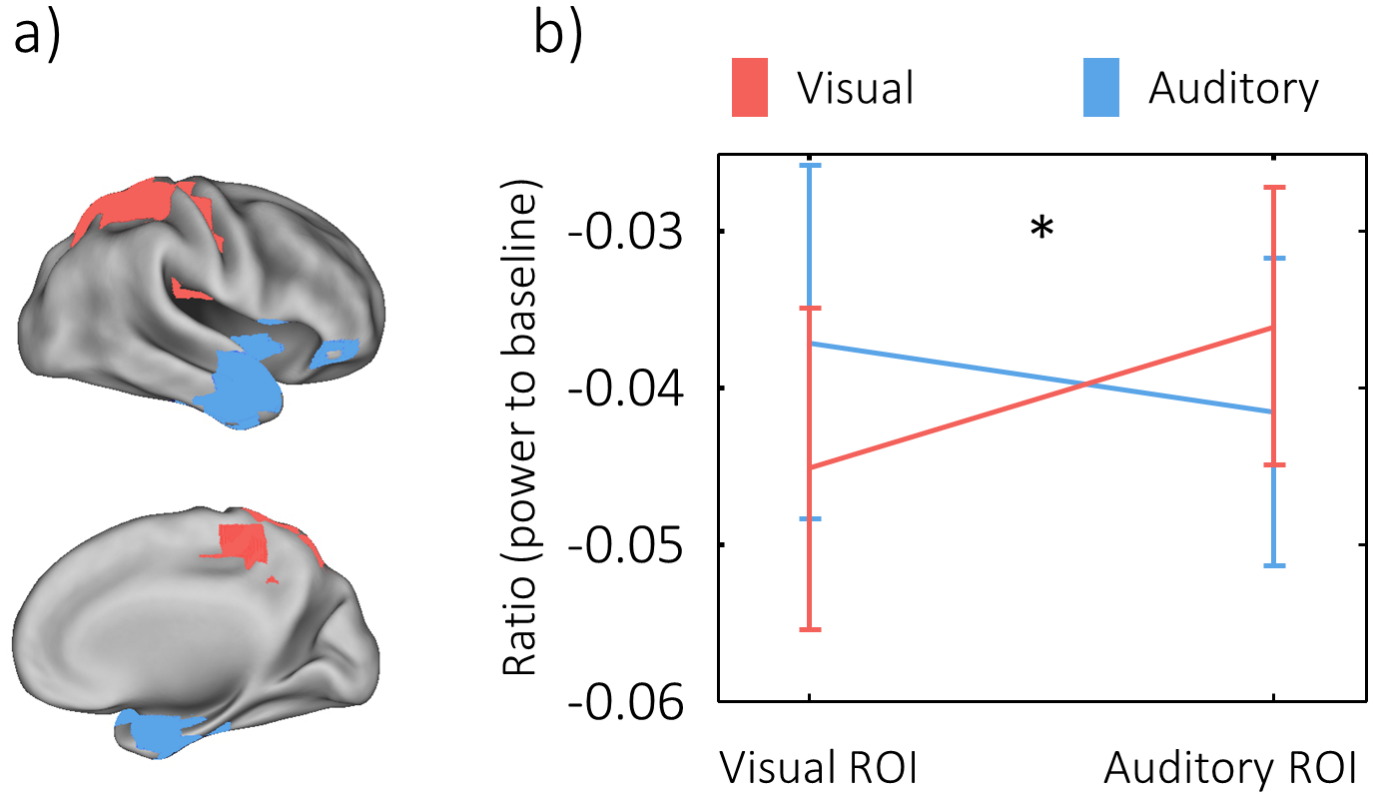
The interaction of power decreases with memory replay. Regions of visual and auditory similarity (a) showed a significant interaction with conditions (*F*_1,23_ = 6.58, *p* = 0.017), such that power decreases in the auditory region of similarity were stronger in the auditory condition, and power decreases in the visual region of similarity were stronger during visual retrieval (b).

Since power decreases at 8 Hz spanned multiple brain regions in the visual and in the auditory condition (compare Fig 3f and 3g), content-specific decreases were still statistically unsubstantiated. In order to link phase-based similarity at 8 Hz with the power decreases during memory replay, we therefore compared the power difference at 8 Hz between hits and correct rejections (see above) within the regions of visual and auditory “replay.” We computed a 2x2 ANOVA contrasting 8 Hz power decreases on the source level, with the factors region (visual/auditory) and condition (visual/auditory). If power decreases are relevant for information coding, stronger power decreases should be observed in those sensory regions where replay occurred. This hypothesis was confirmed by a significant interaction (*F*_1,23_ = 6.58, *p* = 0.017, see Fig 6), showing that power decreases in the auditory region of interest were stronger during replay of auditory memories, whereas power decreases in the visual region of interest were stronger during visual memory replay.

To obtain a further understanding of the temporal dynamics of memory reinstatement, a follow-up analysis within the electrode clusters of significant differences was run for all combinations of retrieval and encoding time windows, resulting in retrieval time–encoding time diagrams (see Fig 5e and 5f). It should be acknowledged that further analyses on this cluster will be biased toward being optimal for the time window on which the electrodes were originally identified. Therefore, the results are likely to show more reinstatement of phase patterns from early encoding. Primarily, this analysis reveals which parts from the original sliding window (centered on the most content-specific cluster from encoding) maximally contributed to the effect when we tested for content specificity of reactivation (e.g., mostly activity from the beginning of the window). Moreover, on a descriptive level, this analysis gives an idea about which phase patterns from encoding, in addition to the early ones, were also reactivated during retrieval.

It is worthwhile to keep two issues in mind when interpreting these plots. First, similarity between two windows will always express temporal smoothing on the diagonal. The diagonal width can be seen as an indicator of the length of the episode that was replayed, but it is also affected by the length of the sliding window (i.e., longer windows will induce more smearing along the diagonal). Second, the peak in these diagonals indicates which temporal pattern at encoding was actually replayed at which retrieval time point. When two time windows are aligned and they share a temporal pattern in their first quarter, this pattern would appear temporally delayed in a one-dimensional plot; however, in two dimensions, we can inspect the diagonal peak of similarity.

In the visual condition, a permutation test revealed significant differences in five clusters. The peaks of the clusters suggested that early, around 141 ms, during retrieval, activity from 672 ms during encoding was reinstated (*p* = 0.017). At 266 ms of the retrieval interval, encoding patterns from around 359 ms reappeared (*p* < 0.002); around 719 ms, phase patterns from 31 ms during encoding were reinstated (*p* = 0.004). Later, during retrieval at 1,859 ms, the phase patterns from 672 ms during encoding were detected (*p* = 0.012), and at 1859 ms, the activity from 1,172 ms during encoding showed a similarity effect (*p* = 0.022).

In the auditory condition, only three clusters could be identified. Peaks within the clusters suggested that 1,203 ms after the onset of the retrieval cue, content from 15 ms during encoding was replayed (*p* = 0.01). Later, at 3,781 ms, activity from 78 ms at encoding reappeared (*p* = 0.017); and finally, at 3,797 ms into the retrieval time, late encoding phase patterns from 1,765 ms could be detected (*p* = 0.014). Even though results are biased toward detecting replay from the early encoding window that served to identify the electrodes on which memory replay took place, this analysis could still give an idea of the temporal dynamics of reinstatement and show the potential of our method. Reactivation of visual patterns was observed very early, as was expected given recent evidence for early reactivation [23,38], and notably earlier than reactivation of auditory patterns, which is in line with a worse memory performance of participants in the auditory condition of this study.

## Discussion

In real life, most of our episodic memories are dynamic with an inherent temporal structure and are not bound to a single modality. We can re-experience information-rich memory traces with auditory and visual content and habitually reinstate these events with an abundance of subjective impressions in their correct temporal order. Although some of these temporal aspects of memory replay have been investigated in spatial navigation experiments in rodents [39–41], the temporal properties of episodic memory replay in humans were largely ignored in previous research. Consequently, little is known about the neural mechanisms that orchestrate the replay of dynamic memories in humans.

In the present study, we identified content-specific temporal signatures of individual memories in the visual and in the auditory domain. These signatures were specific to a carrier frequency of ~8 Hz and could be localized to modality-specific regions, i.e., overlapping with those regions that carried the information of the stimuli during encoding. Strikingly, the 8 Hz frequency also showed the strongest power decrease during retrieval in both modalities. Likewise, the power decrease in 8 Hz during retrieval was modulated in a sensory-specific manner in those regions where memory replay took place, i.e., stronger power decreases in the parietal (visual) region during replay of videos and vice versa for replay of sounds in the temporal (auditory) region (see Fig 6). These findings provide a link to other studies in which a similar interaction between alpha power decreases and oscillatory phase has been proposed to temporally structure perceptual contents [7]. In line with these findings, our results suggest that similar oscillatory mechanisms that guide perception also guide the “re-perception”—that is, memory replay—of these sensory events.

In order to detect the reinstatement of temporal neural patterns that indicate such replay of individual memories, we developed a novel dynamic phase-based RSA method that is robust against variations in the onset of memory replay. This method can therefore be applied in conditions when the exact time point of the reinstatement of a neural pattern is unknown, like, for example, during offline replay in resting state or sleep.

RSA has been previously used to track episodic memories in EEG/MEG [9,22–26] and iEEG [27,28]; however, some important differences to these studies have to be considered. First, we go beyond mere classification of memory content, since we use similarity measures to test a mechanistic hypothesis: that alpha power decreases are associated with the reinstatement of temporal patterns. Hence, we can test whether temporal patterns reappear during retrieval, and we can link this replay to a specific frequency band. Importantly, the detection of temporal patterns was only made possible with our dynamic RSA approach.

Second, in our design, we carefully avoided any sensory overlap between encoding and retrieval. We were therefore able to investigate mechanisms of purely memory-driven reinstatement, as opposed to studies in which there was a high overlap in sensory stimulation between encoding and retrieval [25,28,42]. This aspect of our experimental design allows us to conclude that the brain actively reproduces a temporal pattern that is specific to a stimulus in order to re-experience this particular memory.

An important open question concerns how the hippocampus is involved in the replay of temporal patterns in the cortex, as observed here. A critical involvement of the hippocampus, and the phase of theta oscillations therein, for memory replay is implicated by recent models and frameworks [8,43,44]. Future studies are required that record simultaneously from both the hippocampus and the neocortex to investigate how the reinstatement of the temporal phase patterns described here interacts with, or relies on, the hippocampus.

Studying the temporal aspects of memory replay has proven to be difficult, because methods or stimulus material in previous studies did not allow for investigating this question. Overcoming these previous limitations, we identified a potential domain general mechanism that orchestrates the replay of dynamic auditory and visual memories in humans. Specifically, our findings suggest an intimate relationship between power decreases in an 8 Hz frequency and a content-specific temporal code, carried by its phase. These results corroborate recent theories linking power decreases with the coding of neural information [8,45,46]. Our findings open up new ways of investigating the temporal properties of memory replay in humans, which we only begin to understand.

## Materials and Methods

Ethical approval was granted by the University of Birmingham Research Ethics Committee (ERN_14–0651), complying with the Declaration of Helsinki.

### Participants

Twenty-four healthy, right-handed subjects (18 female and 6 male) volunteered to participate. Seven further participants were tested, or partly tested, but could not be analyzed due to poor memory performance *(n* = 2), misunderstanding of instructions (*n* = 2), and poor quality of EEG-recording and technical failure (*n* = 3). All participants had normal or corrected-to-normal vision. The average age of the sample was 23.38 (s.d. = 3.08) years. Participants were native English speakers (20), bilingual speakers (2), or had lived for more than 8 y in the United Kingdom (2). Participants provided informed consent and were given a financial compensation of £24 or course credit for participating in the study.

### Material and Experimental Setup

The cues amounted to 360 words that were downloaded from the MRC Psycholinguistic Database [47]. Stimulus material consisted of four video clips and four sound clips in the visual and auditory session, respectively. All clips were 3 s long; videos showed colored neutral sceneries with an inherent temporal dynamic, and sounds were short musical samples, each played by a distinct instrument. In both sessions, a clip was associated with 30 different words. Sixty words were reserved for the distractor trials, and 12 additional words were used for instruction and practice of the task. For presentation, words were assigned to the clips or to distractors in a pseudorandom procedure, such that they were balanced for Kucera-Francis written frequency (mean = 23.41, s.d. = 11.21), concreteness (mean = 571, s.d. = 36), imageability (mean = 563.7 s.d. = 43.86), number of syllables (mean = 1.55, s.d. = 0.61), and number of letters (mean = 5.39, s.d. = 1.24). Furthermore, lists were balanced for word frequencies taken from SUBTLEXus [48]. Specifically, “Subtlwf” was employed (mean = 20.67, s.d. = 27.16). The order of presentation was also randomized, assuring that neither the clips and their associates nor distractor words were presented more than three times in a row or in temporal clusters. The presentation of visual content was realized on a 15.6-in CRT monitor (Taxan ergovision 735 TC0 99) at a distance of approximately 50 cm from the subject’s eyes. The monitor refreshed at a rate of 75 Hz. On a screen size of 1,280 x 1,024 pixels, the video clips appeared in the dimension of 360 pixels in width and 288 pixels in height. “Arial” was chosen as the general text font, but font size was larger during presentation of word-cues (48) than during instructions (26). In order to reduce the contrast, white text (rgb: 255, 255, 255) was presented against a grey background (rgb: 128, 128, 128). Auditory stimuli were presented using a speaker system (SONY SRS-SP1000). The two speakers were positioned at a distance of approximately 1.5 m in front of the subject, with 60 cm of distance between the speakers.

### Procedure

Upon informed consent and after being set up with the EEG-system, participants were presented with the instructions on the screen. Half of the subjects started with the auditory session; the others were assigned to undertake the visual task first. Both sessions consisted of a learning block, a distractor block, and a test block. The sessions were identical in terms of instructions and timing and differed only in the stimulus material that was used. During instruction, the stimulus material was first presented for familiarization and then used in combination with the example words to practice the task. Instructions and practice rounds were completed in both sessions.

As a way to enhance memory performance, participants were encouraged to use memory strategies. The suggestion was to imagine the word in a vivid interaction with the material content, yet the choice of strategy remained with the subject. In the learning block, 120 clip-word sequences were presented. Each sequence started with a fixation cross that was presented in the center of the screen for 1 s, then the video clip played for 3 s. In the auditory condition, the fixation cross stayed on the screen and the sound clip played for 3 s. Immediately after the clip, a word cue was presented in the center for 4 s, giving the subject time to learn the association. After that, an instruction asked the subject to subjectively rate on a six-point scale how easy the association between the clip and the word was. After a press on the space bar, this scale was shown. Equidistant categories were anchored with the labels “very easy” and “very hard;” those labels were displayed at both ends above the scale. Participants used six response buttons to rate the current association (see Fig 1).

In the distractor block, subjects engaged in a short, unrelated working memory task; namely, they counted down in steps of 13, beginning from 408 or 402, respectively. After 1 min, the distractor task ended. Following a short self-paced break, subjects refreshed the instructions on the retrieval block.

In this block, either a cue or a distractor was presented upon a button press on the space bar. Subjects were instructed to try to vividly replay the content of the corresponding video clip or sound -clip in their mind upon presentation of the cue. The word stayed on the screen for 4 s, giving the subject the opportunity to replay the memory. Finally, a fixation cross was presented for a varying time window between 250 and 750 ms, to account for movement and preparatory artifacts, before the response scale appeared on the screen.

The response scale consisted of six options. Four small screenshots of the videos or four black-and-white pictures of the featured instruments were presented in equidistant small squares of 30 x 30 pixels. Additionally, the options “new” and “old” were displayed in the form of text at the most left and most right position of the scale (see Fig 1c and 1d). Subjects could now indicate the target (video/sound) they just replayed by pressing the button corresponding to that clip. In addition, subjects could also indicate that the word was a distractor by pressing the button corresponding to the option “new,” or they would simply indicate that they remembered the word, but could not remember the clip it was associated with. In this last scenario, subjects would press the button corresponding to “old.” The positions of “old” and “new” at the end of the scale, as well as the permutation of the four target positions in the middle of the scale, were counterbalanced across participants. Finally, after making a decision, a further six-point rating scale was presented on which subjects could rate their confidence in their response. Again, a scale with equidistant categories was presented, ranging from “guess” to “very sure.” An additional possibility was to press “F2” in case of an accidental wrong button press. In this case, the whole trial was discarded from analysis. Following the retrieval block, individual electrode positions were logged, allowing for a break of approximately 30 min before beginning the second session. In addition to the two experimental sessions, all participants came to a separate session to record anatomical MRI scans at the Birmingham University Imaging Centre (https://www.buic.bham.ac.uk/). This was later used to facilitate source localization (see below).

### Data Collection

The recording of behavioral responses and the presentation of instructions and stimuli were realized using Psychophysics Toolbox Version 3 [49] with MATLAB 2014b (MathWorks) running under Windows 7, 64 Bit version on a desktop computer. Response buttons were “s, d, f, j, k, l” on a standard “QWERTY” layout. Buttons were highlighted and corresponded spatially to the response options on the screen, so participants didn’t have to memorize the keys. To this end, the shape of corresponding fingers was also displayed under the scale. To proceed, participants used the space bar during the experiment. Physiological responses were measured with 128 sintered Ag/AgCl active electrodes, using a BioSemi Active-Two amplifier. The signal was recorded at a 1,024 Hz sampling rate on a second computer via ActiView recording software, provided by the manufacturer (BioSemi, Amsterdam, the Netherlands). Anatomical data was acquired using magnetic resonance imaging (MRI) (3T Achieva scanner; Philips, Eindhoven, the Netherlands), and electrode positions were logged with a Polhemus FASTRAK device (Colchester, Vermont, USA) in combination with Brainstorm [50] implemented in MATLAB.

### Preprocessing

The data was preprocessed using the Fieldtrip toolbox for EEG/MEG-analysis [51]. Data was cut into trial-segments from 2 s pre-stimulus to 4.5 s after stimulus onset (i.e., onset of the clip at encoding and onset of the word at retrieval). The linear trend was removed from each trial, and a baseline correction was applied based on the whole trial. Trials were then downsampled to 512 Hz, and a band-stop filter was applied at 48–52, 58–62, 98–102, and 118–122 Hz to reduce line noise at 50 Hz and noise at 60 Hz; additionally, a low-pass filter at 140 Hz was applied. After visual inspection for coarse artifacts, an independent component analysis was computed. Eye-blink artifacts and eventual heartbeat/pulse artifacts were removed, bad channels were interpolated, and the data was referenced to average. Finally, the data was inspected visually and trials that still contained artifacts were removed manually. MRI scans of each participant were segmented into four layers (brain, cerebrospinal fluid, skull, and scalp) using SPM8 (http://www.fil.ion.ucl.ac.uk/spm/) in combination with the Huang toolbox [52]. On this basis, a volume conduction model was created with the Fieldtrip “dipoli” method; individual electrode positions were aligned to the head model for every participant.

### Behavioral Analysis

For behavioral analysis, correct trials were defined as those of the retrieval phase in which the target was correctly identified and the confidence rating of the response was high (5 or 6). Trials were defined as correct rejections if a distractor word was correctly identified as new; misses were defined as trials in which a cue word was incorrectly identified as a new word or the response “old” was given to indicate that the subject recognized the word, but could not remember the target video or sound it was associated with. Hits of low confidence were not considered in subsequent analyses. Furthermore, selections of the wrong clip as well as accidental presses of the wrong button and distractor trials that were not recognized as distractors were discarded from further analysis.

### Power Analysis

Power at retrieval was determined by multiplying the Fourier-transformed data with a complex Morlet wavelet of six cycles. Raw power was defined as the squared amplitude of the complex Fourier spectrum and estimated for every fourth sampling point (i.e., sampling rate of 128 Hz). For each contrast (i.e., hits versus misses, or hits versus correct rejections), baseline normalization was performed separately. Therefore, a baseline was computed as the average power between -1 and 4 s of all trials within the contrast [15]. Every trial was then normalized by subtracting the baseline and subsequently dividing by the baseline (activity_tf_−baseline_f_)/baseline_f_, where t indexes time and f indexes frequency. The relative power was calculated for all frequencies between 1 and 40 Hz.

### Phase Stationarity

**Fig 7:**
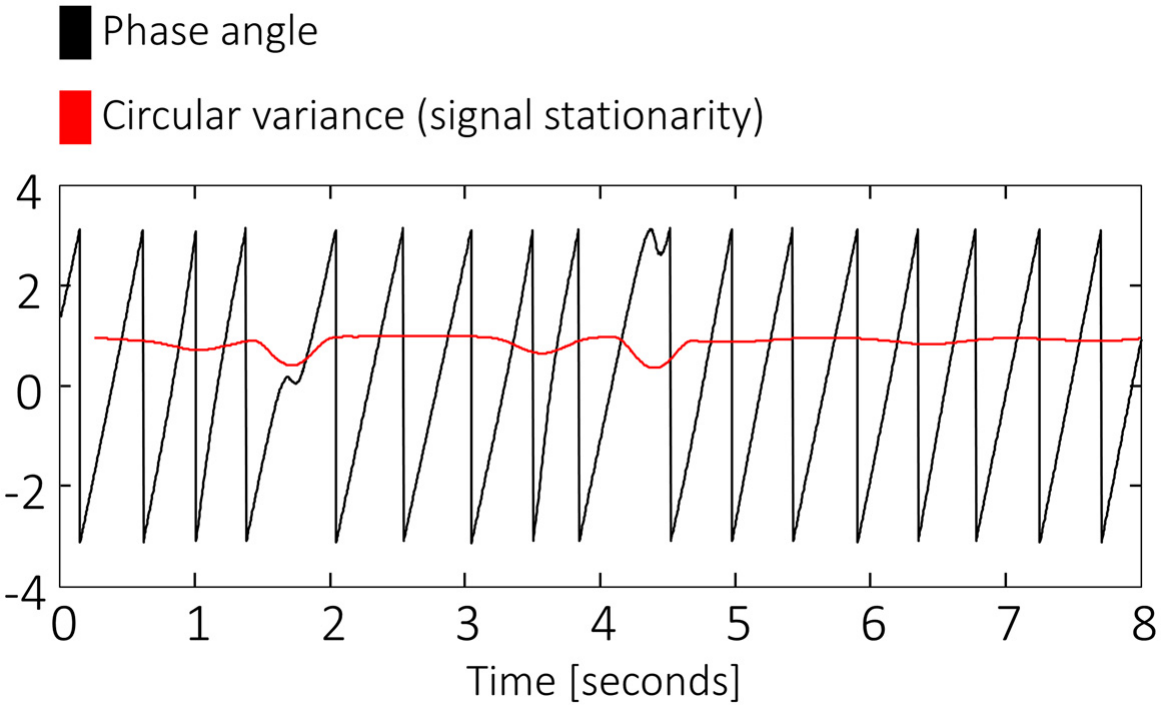
Circular variance within a sliding window of one cycle as a measure of signal stationarity. When the signal is stationary and there are no phase resets, the circular variance reaches 1.

For every frequency between 2 and 40 Hz, the stationarity of phase was defined within a sliding window of one cycle (see S2 Fig, S2 Text, S3 Fig, S3 Text, S4 Fig). Phase was estimated by multiplying the Fourier-transformed data with a complex Morlet wavelet of six cycles. The complex signal was then divided by its amplitude to standardize its power to 1. At every time point, the deviation from an even circular distribution within one cycle around this point was assessed, i.e., the circular variance (CV) of phase over time was computed. CV was interpreted as a measure of signal stationarity, since a perfectly stationary signal has an even distribution over one cycle and the circular variance within the cycle is maximal (i.e., reaches 1; see Fig 7). Phase stationarity was baseline corrected in the same way as oscillatory power. A baseline was computed as the average stationarity between -1 and 4 s of all trials within the contrast. Every trial was then normalized again by subtracting the baseline and then dividing by the baseline (stationarity_tf_−baseline_f_)/baseline_f_, where t indexes time and f indexes frequency.

### Content Specificity of Phase at Encoding

While participants learned the associations in the encoding block, they repeatedly saw (heard) the same dynamic stimulus. Content-specific properties could consequently be identified if they were shared by trials of the same content but not by trials of a different content.

Hence, content-specific phase was assessed by contrasting the phase similarity between pairs of trials in which the same content was presented, with the phase similarity of an equal number of trial pairs that were of different content. To achieve this, trials were grouped and combined in a random but balanced way (see below). For each pair of trials, the cosine of the absolute angular distance was then computed and finally averaged across all (same or different) combinations [29].

This resulted in an average similarity value at every time point, at every electrode and in every frequency of interest. This similarity was derived separately for the same pairs and for the different pairs and could consequently be subjected to statistical testing in order to define content specificity of phase.

**Fig 8:**
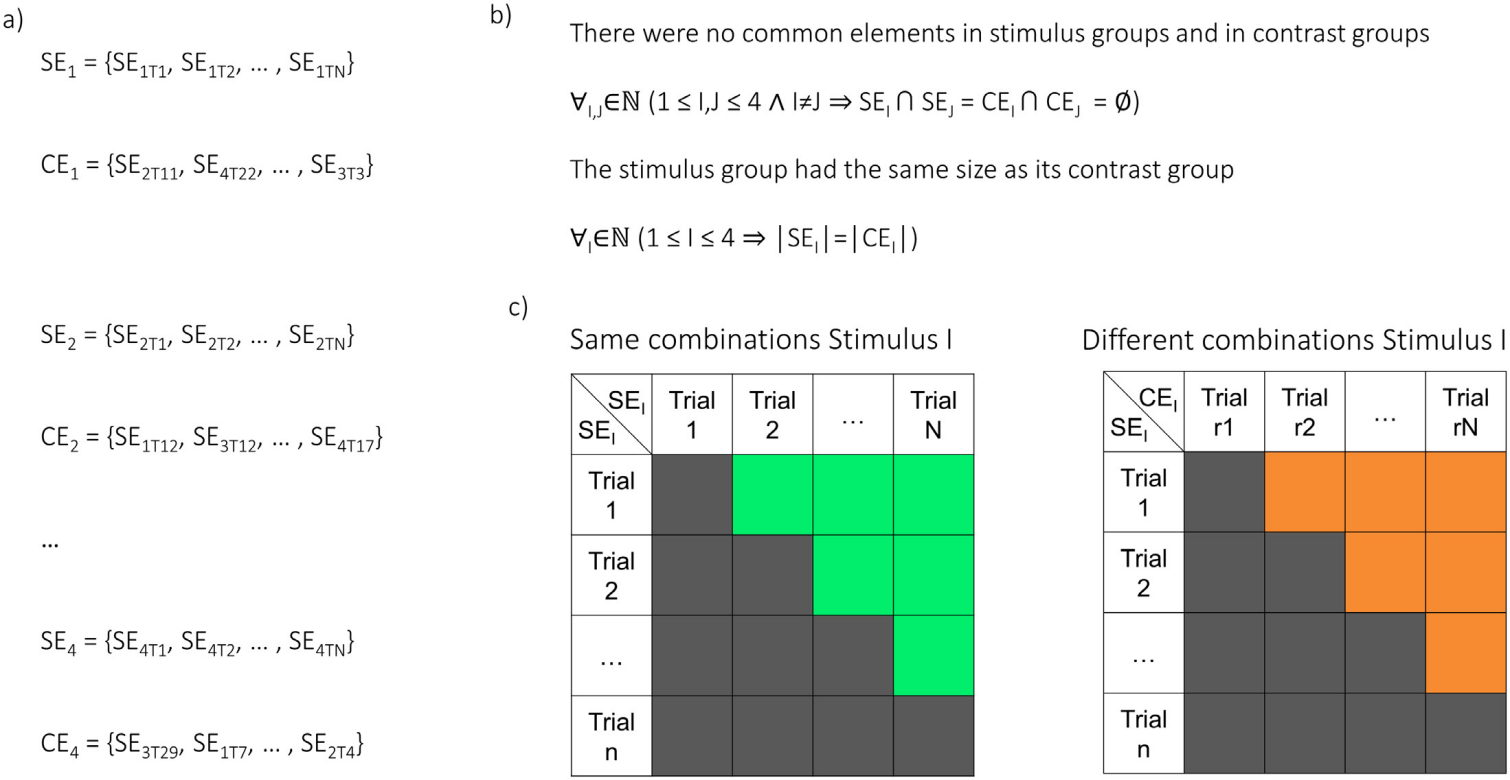
Trial combinations between same and different content during encoding. Each cell in the upper right regions (green and orange) indicates a pair of trials that are compared, i.e., cosine of angular distance. SEk,Tm denotes a trial, where k denotes one of the four videos (or sounds), and m a trial of that video (or sound). (a) Trials at encoding were divided into sets of the same stimulus content (SE1-4). A contrast set, namely, a set containing a random selection of trials of different content, was assigned to every stimulus set (CE1-4). (b) None of the same-content sets or the contrast sets shared any trial. The size of each stimulus set was the same as the size of its contrast set. To ensure this, some trials were discarded from analysis. (c) For N trials of content I (in the set SEI), the N*(N-1)/2 unique trial combinations were built. This corresponds to the above diagonal region of a combination matrix (green cells). From all the possible combinations of different content between a trial set (SEI) and its contrast set (CEI) only the combinations above the diagonal were selected (orange cells) for contrast. This is equivalent to exchanging one side of the combinatory pairs that were built within a stimulus set (i.e., left matrix), with trials from its contrast set (replacing all instances of one trial with instances of a trial from the contrast set). The same-content combinations and the different-content combinations of all four stimuli were then contrasted.

Importantly, the way of combining the trials can result in bias. For this reason, the trial combinations were randomly selected in a carefully balanced way (Fig 8). Firstly, the trials were grouped into four sets that were of the same content (SE_1-4_), e.g., the same video. These sets were then recombined such that each set of content, say, A, could be paired with a unique set of mixed content (say, B, C, and D) that was equal in size, i.e., a contrast-set (CE_1-4_). To make this possible, some trials were discarded from further analysis (Fig 8a and 8b).

In order to form pairs of same content, all possible N*(N-1)/2 pairs within each of the four stimulus-sets (SE_1-4_) were built. Then, to form pairs of different content, only N*(N-1)/2 pairs between the stimulus-set (SE_I_) and its contrast-set (CE_I_) were built. Importantly, wherever the second trial in the pairs of same content appeared in several combinations, it was replaced by instances of the same exclusive trial from the contrast set, while building the combinations of different content (Fig 8c).

### Content-Specific Phase Similarity between Encoding and Retrieval

Participants not only saw (heard) the same dynamic stimulus several times in the encoding block; they also repeatedly recalled the same memory content. This made it possible to detect content-specific properties of memories if they were shared by trials in which the same content was learned and remembered (e.g., encoding A, remembering A) but not by trials in which different content was learned and remembered (e.g., encoding B, remembering C). Content-specific phase was consequently assessed by contrasting the phase similarity between encoding-retrieval pairs of same content, with the phase similarity of encoding-retrieval pairs that were of different content.

**Fig 9:**
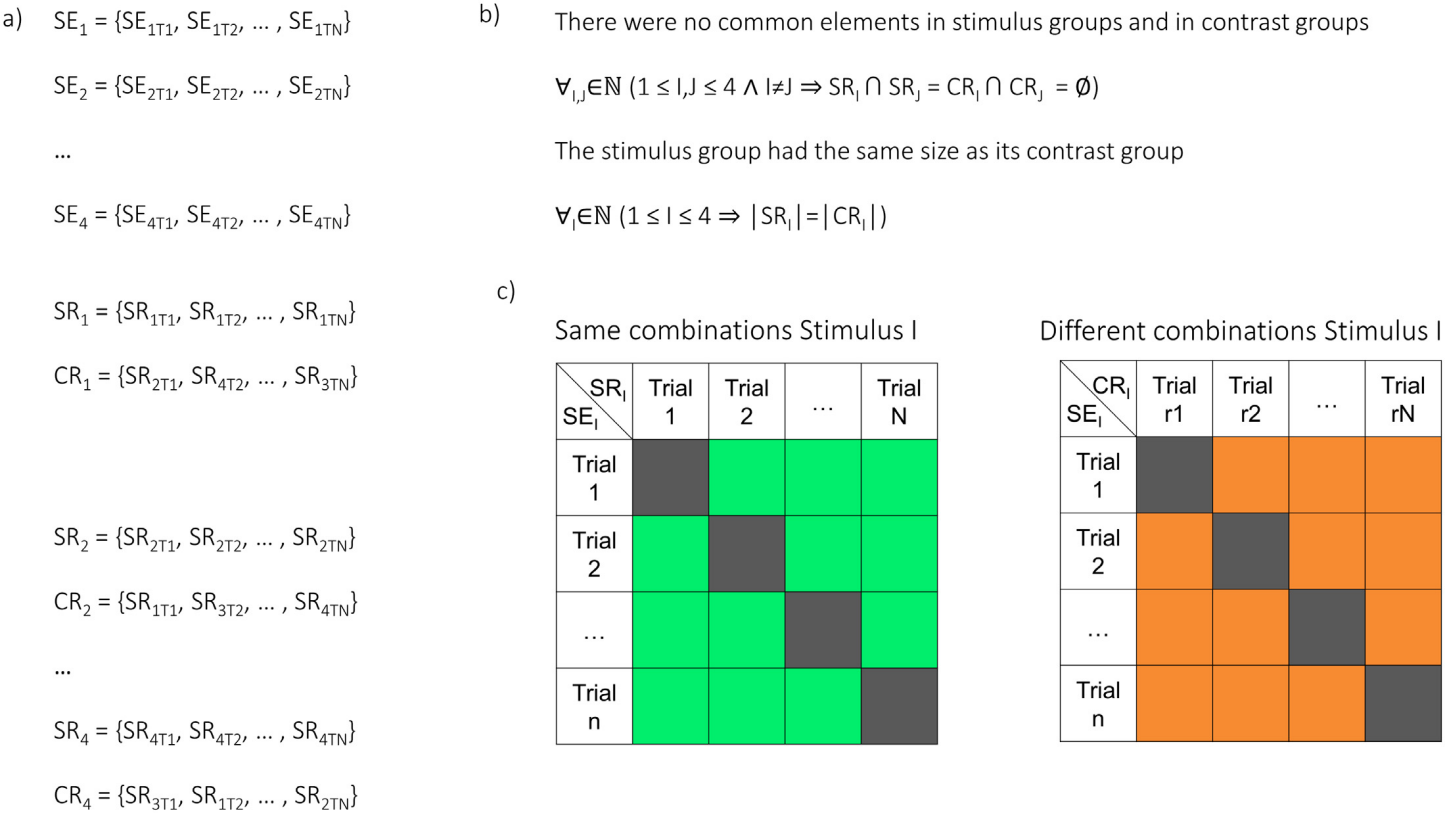
Trial combinations of same and different content between encoding and retrieval. (a) Trials were divided into sets of the same stimulus content at encoding (SE_1-4_) and at retrieval (SR_1-4_). Stimulus content at retrieval refers to the content held in memory. A contrast set (CR_1-4_), namely a set containing a random selection of trials of different content, was assigned to every stimulus set (SR_1-4_) at retrieval. (b) The different stimulus sets at retrieval (SR_1-4_) as well as the different contrast sets (CR_1-4_) had no common trials. Furthermore, every stimulus set at retrieval had the same number of trials as its contrast set. To ensure this, some trials were discarded before further analysis. (c) The combinations of same content between encoding and retrieval consisted of all possible trial pairs between a set of content I at encoding (SE_I_) and the set of content I at retrieval (SR_I_). However, combinations containing the same word cue were ignored (diagonal grey cells). Combinations of same content therefore correspond to the off-diagonal of a combinatory matrix (green cells). To build the combinations of different content for a stimulus, the trials from the very same set at encoding (SE_I_) were then combined with all trials from the corresponding contrast-set to its content at retrieval (CR_I_). Combinations on the diagonal were ignored accordingly; different content combinations correspond to the off-diagonal of the combinatory matrix (orange cells). The similarity of same-content pairs was finally contrasted with the similarity of different-content pairs for all four dynamic stimuli.

Again, trials were grouped and paired in a balanced randomization procedure to avoid potential bias. First, the trials at encoding were grouped into four sets that were of same content (SE_1-4_). Likewise, the trials at retrieval were grouped into four sets of same memory content (SR_1-4_). These sets at retrieval were then recombined, such that each set of content A could be assigned a unique set of mixed content (B, C, and D) that was equal in size, i.e., a contrast set (CR_1-4_). To make this possible, some trials were discarded from further analysis (Fig 9a and 9b).

Encoding-retrieval pairs of same content were then formed by building all possible pairs of trials between each set of a content at encoding (SE_I_) and the corresponding set of this memory content at retrieval (SR_I_). In order to build the pairs of different content, the very same set of trials from encoding (SE_I_) was combined with the corresponding contrast set (CR_I_) at retrieval. Finally, pairs containing the same word cue were ignored. This occurs when the encoding trial that was originally associated with a word cue was combined with the retrieval trial in which this cue was actually presented. Accordingly, in the combinations of different content, the pair between the discarded encoding trial and a random trial was ignored (Fig 9c).

Between the pairs of same combinations, a similarity measure of phase was then computed (see below) and contrasted with the similarity between the pairs of different content. In order to maximize the signal-to-noise ratio in further analysis, several restrictions were applied to define frequencies, time windows, and electrodes of interest. The tested frequency was 8 Hz, because both conditions expressed the strongest correlates of memory in this frequency band. Furthermore, the time window at encoding was restricted to a 1-second episode in which phase patterns were maximally different between the stimuli. The window was defined around the center of the cluster in which phase patterns were most reliably content-specific during encoding (i.e., the cluster with the lowest *p*-value). Centering the encoding window on the most content specific time course of activity should increase the sensitivity to detect differences from encoding at retrieval. Likewise, the electrodes for further analysis were restricted to the electrodes within that cluster (128/128 electrodes in the visual condition and 107/128 electrodes in the auditory condition). It needs to be emphasized that none of these restrictions leads to circular inference, because all of these prior restrictions are independent of the similarity between encoding and retrieval trials. Most importantly, phase similarity at encoding, under the null hypothesis, is completely orthogonal to any neural activity at retrieval.

Phase similarity between two windows was then assessed with the Single-trial Phase Locking Value (S-PLV) [32,33]. This measure defines similarity between two windows (x and y) as the constancy of phase angle difference over time, where n denotes the width of the window and *φ* is the phase:

$$SPLV\;=\;\left|n^{-1}\sum_{t\;=\;1}^{n}e^{i(\varphi xt-\varphi yt)}\right|$$

If the two signals are very similar over time, the phase angle differences will not vary much (i.e., have low circular variance). In this way, the similarity of two windows can be quantified as 1 minus the circular variance of phase differences over time. S-PLV has the advantage of increased robustness for noisy data at the expense of temporal resolution. For the purposes of assessing similarity between two oscillatory patterns, this measure is convenient because it affords a high degree of temporal invariance and results in a value between 0 and 1 when two oscillatory patterns are compared. Therefore, despite the oscillatory nature of temporal patterns in the EEG, this makes it possible to assess the average similarity across time, trials, and subjects. In their paper, the authors suggest computing the S-PLV over 6–10 cycles of a frequency for a good signal-to-noise ratio [32]; for our purposes, S-PLV was applied to a time window of 8 cycles, which resulted in a 1-second window for 8 Hz. Phase values were extracted by multiplying the Fourier-transformed data with a complex Morlet wavelet of six cycles. Phase values were then downsampled to 64 Hz. The similarity measure was computed for every pair of trials in the combinations of same content and in the combinations of different content. Importantly, a sliding window approach was used to account for the non-time-locked nature of the data (memory reactivation could happen at any time during retrieval).

For every combination of trials, this resulted in a single similarity value for every electrode and every time point at retrieval, i.e., the similarity to the 1-second encoding window (a similarity value at a single time point represented the similarity of the surrounding 1-second window at retrieval, to that window from encoding). Additionally, the retrieval window was truncated at 4 s in order to avoid potential confounds from post-stimulus images or responses; to assess similarity at 4 s, the time window was instead continued beginning from 1 s pre-stimulus (i.e., similarity at 4 s reflects the similarity between the encoding window and the concatenated window from 3,500 ms to 4,000 ms and -1,000 ms to -500 ms at retrieval).

In order to test for content-specific phase patterns, the difference in similarity between same-content combinations and different-content combinations was averaged across the whole retrieval episode (between 0 and 4,000 ms), which resulted in a single value for every electrode for the same content combinations and for the different content combinations. Those values were then statistically tested across subjects, controlling for multiple comparisons with the fieldtrip permutation procedure [53]. Additionally, two control frequencies were tested that were approximately in the golden mean ratio (i.e., maximally different in terms of phase) to 8 Hz [34], namely 5 and 13 Hz. Two further control frequencies were tested that showed the next strongest power decrease in one of the conditions, namely 4 and 15 Hz. Encoding time windows were defined accordingly for these frequencies as eight cycles around the center of the most reliable cluster during encoding.

The electrode clusters of significant differences that resulted for 8 Hz were subjected to further analysis in order to explore the temporal dynamics of reinstatement. In a first step, a series of post-hoc *t*-tests was computed on the difference between same and different content combinations during every time point of retrieval. This resulted in a time series that is comparable to a cross-correlogram and can be interpreted as a time course of reinstatement (see Fig 5a and 5b).

In a further step, the sliding window analysis was repeated with different time windows from encoding, but keeping with the electrodes in the cluster of significant differences. Thereby, similarity between any two time points could be estimated with a temporal uncertainty of +/-500 ms. The outcome of this analysis was a matrix of similarity between every time point at encoding and every time point at retrieval on each of the electrodes in the cluster (see Fig 5e and 5f). The difference between combinations of same and different content was then averaged across electrodes and tested over subjects. The resulting clusters reveal the temporal relationship between presentation at encoding and reinstatement during retrieval; however, it should be said that tests on this encoding-retrieval matrix are not independent from the original identification of the electrodes.

### Source Reconstruction

To reconstruct the activity on the source level, a linearly constrained minimum variance (lcmv) beamforming approach was used, as it is implemented in the Fieldtrip toolbox (http://www.fieldtriptoolbox.org/). Individual electrode positions were used together with boundary element models that were constructed from individual MRI scans. With lcmv-beamforming, filters will be more accurate for the data that they were constructed on and will also be more accurate if constructed on a long time interval [54]. This trade-off was addressed by computing each filter around the preprocessed data that contributed to the effect being localized. Power differences were localized with a filter based on -500 ms to 4,500 ms at retrieval; for the phase similarity at encoding, the filters were estimated on the time window between -500 ms and 3,500 ms of the encoding trials. Phase similarity between encoding and retrieval was reconstructed with a filter based on -500 ms to 1,000 ms at encoding and -500 ms and 4,500 ms at retrieval. Activity on 2,020 virtual electrodes was thereby reconstructed and the analysis of the data was repeated in the same way on the virtual data.

### Statistical Analyses

#### Behavioral performance

Behavioral results were compared between the auditory and visual condition with a series of paired *t*-tests. *P*-values were compared against a Bonferroni-corrected threshold [55]; however, no specific hypothesis was tested.

#### Decreases in power

To test for differences in baseline corrected power, a paired *t*-test was first computed for every time point and frequency at every channel. For multiple-comparison correction, a random permutation procedure was applied [53]. This procedure sums up neighboring *t*-values above a cluster-forming threshold and compares the resulting clusters’ sizes to the distribution of the maximal cluster sums that are derived when condition labels are randomly swapped with the Monte-Carlo method. The option for the minimum number of channels to be considered a cluster was specified with three. This attenuates the confound coming from spatially high frequency noise only allowing clusters that contain at least three neighboring channels above threshold; neighboring electrodes were derived via the triangulation method of the Fieldtrip toolbox (http://www.fieldtriptoolbox.org/). The clusters were summed across time, frequency, and channels, and labels were then permuted 1,000 times; computation of the clusters as well as the testing of the null hypothesis was addressed with a threshold for two-sided testing (alpha level of 0.025). Due to computational limitations, the power values were downsampled before the unrestricted test across time, electrodes, and frequencies, such that values were included approximately every 16 ms. To further identify frequencies with a reliable power difference, a paired samples *t*-test was computed for every frequency on the average power difference across channels and across the whole retrieval time window between 0 and 4 s (for frequencies below 6 Hz, the time window was increasingly shorter, since the last point of data was at 4.5 s, and the power of lower frequencies cannot be estimated toward the boundaries of the time window).

On the source level, the average power values between 0 and 4 s were compared for 8 Hz. Therefore, a *t*-test was computed for every virtual electrode and an unrestricted permutation procedure was run on the whole brain level in the same way as described above, using 1,000 permutations. Neighboring *t*-values were now only spatially defined from neighboring virtual electrodes.

#### Phase stationarity/signal complexity

In order to assess whether the frequency-specific power decreases resulted in differences in phase stationarity, a paired *t*-test was computed for the average difference in stationarity over time and electrodes (see S2 Fig, S2 Text, S4 Fig, S4 Text). These values were averaged over all data points in the time window between 0 and half a cycle before the end of the trial (4 s). Following the hypothesized dependency of phase stationarity on the power decreases, the frequency with the strongest power decrease was tested first. In subsequent tests, *p*-values were compared against a Bonferroni-corrected *p*-value.

#### Phase similarity during encoding

Phase similarity at encoding was tested in the same way as power. A series of paired *t*-tests was computed to contrast the average similarity of combinations of same content with the average similarity of combinations of different content. *T*-values for every frequency band, electrode, and time point were then corrected for multiple comparisons in an unrestricted cluster-based permutation approach. The cluster permutation compared again the sums of *t*-values across frequency, electrodes, and time against the distribution of these clusters derived via the Monte-Carlo method. Due to computational limitations, the similarity values were downsampled only for the unrestricted test across time, electrodes, and frequencies, such that approximately every 16 ms a value was included in the test. A threshold for two-sided testing was applied in order to test against the null hypothesis. Later, the frequency 8 Hz was tested separately with the same cluster permutation procedure against a one-sided threshold in order to identify a temporospatial cluster, in which 8 Hz phase could differentiate content particularly well. On the source level, similarity was averaged over the defined 1-second encoding window (see above) and contrasted between combinations of same and different content with a *t*-test on every virtual electrode. Multiple comparisons correction was performed again on the whole brain level, neighboring *t*-values were summed, and the distribution of resulting clusters was created using 1,000 randomly drawn permutations. The probability of the observed cluster was then assessed by comparing its size to this distribution, correcting for a threshold of two-sided testing.

#### Phase similarity between encoding and retrieval

The similarity between the encoding window and the retrieval episode was tested for differences between combinations of replay of the same versus replay of different content. In a first step, the average difference between 0 and 4 s was contrasted with a paired *t*-test on every electrode to test for a general effect. For multiple-comparison correction, again, 1,000 permutations were drawn and observed clusters of summed *t*-values were tested against the distribution of sums under random permutation of conditions; the threshold for significance was the *p*-value for two-sided testing. The test of the effect on the source level was small and did not survive multiple-comparison correction. However, the maximal clusters of neighboring *t*-values that exceeded a threshold for single-sided testing were assumed to reflect the effect that was significant on the electrode level. Therefore, we used only the maximal cluster of differences in source space as a region of interest for further analyses.

#### Interaction of power decreases and phase similarity in source space

These clusters of similarity were then tested for differences in power decreases by summing up power differences across all virtual electrodes within each region of interest (visual/auditory) in each condition (visual/auditory). Power in these regions was then compared across conditions by subjecting the power decreases in the regions to a 2 x 2 repeated measurements ANOVA with the factors *region of interest* and *condition*.

#### Exploration of encoding retrieval similarity in the cluster of memory replay

Finally, on the electrode level, the similarity effect between encoding and retrieval was statistically explored. Within the electrode clusters of significant differences, a series of post-hoc *t*-tests was computed and thresholded with a probability level of 0.01 for a single-sided test in order to identify the time windows that caused the similarity effect. Lastly, the similarity matrices within the clusters of electrodes that indicated encoding-retrieval similarity were tested. Neighboring *t*-values of difference between same and different content combinations were thresholded again at a *p*-value of 0.01 and summed up; however, in order to allow for negative effects and rule them out, the threshold was adapted for two-sided testing. One thousand permutations were drawn, and a distribution of the strongest clusters, second strongest clusters, etc. was built. The observed clusters were sorted and compared against the random distribution of clusters. A liberal approach was adopted, comparing the cluster with the highest sum of *t*-values, to the distribution of the maximal cluster and every following cluster to the distribution of next strongest clusters. Critical *p*-values for significance were divided by the number of the cluster [55].

## Data Availability

Group statistical data and analysis scripts of this project are deposited in the Dryad repository: http://dx.doi:10.5061/dryad.ch110 [56].

## Supporting Information

S1 Fig Contrast of hits and misses. Successful memory reinstatement was associated with a cluster of broad power decreases in the lower frequencies (<30 Hz). (a-b) Sum of *t*-values across the electrodes in the cluster of significant differences for the visual condition (a) and for the auditory condition (b). (c) *T*-statistic of power decrease, averaged over electrodes and time. (d,e) Topography of power decreases in the visual condition (d) and in the auditory condition (e). Power decreases are plotted as *t*-values of average difference at 8 Hz between 0 and 4 s during retrieval. (f,g) Reconstruction of 8 Hz power difference in source space using an “lcmv” beamforming-algorithm in the visual (f) and in the auditory condition (g).
(TIF)

S2 Fig Stationarity, hits minus correct rejections. Successful memory reinstatement was associated with decreases in the stationarity of the signal. In the contrast of hits and correct rejections, the decrease peaked at 8 Hz (a). Topographies of differences in 8 Hz stationarity are shown on the right in the visual (b) and in the auditory condition (c).
(TIF)

S3 Fig Stationarity, hits minus misses. Successful memory reinstatement was associated with decreases in the stationarity of the signal. In the contrast of hits and misses, the decrease peaked at 8 Hz (a). Topographies of differences in 8 Hz stationarity are shown on the right in the visual (b) and in the auditory condition (c).
(TIF)

S4 Fig Simulation of the relationship between signal amplitude, signal complexity (1-circular variance) and noise. The complexity of the signal with lower amplitude is more strongly affected by noise.
(TIF)

S1 Movie One of the four movies that were used as stimuli. (MP4)

S2 Movie One of the four movies that were used as stimuli. (MP4)

S3 Movie One of the four movies that were used as stimuli. (MP4)

S4 Movie One of the four movies that were used as stimuli. (MP4)

S1 Sound One of the four sounds that were used as stimuli. Note that volume was adjusted for presentation.
(WAV)

S2 Sound One of the four sounds that were used as stimuli. Note that volume was adjusted for presentation.
(WAV)

S3 Sound One of the four sounds that were used as stimuli. Note that volume was adjusted for presentation.
(WAV)

S4 Sound One of the four sounds that were used as stimuli. Note that volume was adjusted for presentation.
(WAV)

## Abbreviations

BOLD: blood-oxygen-level dependent
EEG: electroencephalographic
fMRI: functional magnetic resonance imaging
iEEG: intracranial EEG
PPC: pairwise phase consistency
RSA: representational similarity analysis
S-PLV: single-trial phase locking value
