# Supplementary figures and images for: The Temporal Signature of Memories: Identification of a General Mechanism for Dynamic Memory Replay in Humans
Source: PLoS Biol. 2016 Aug 5;14(8):e1002528. doi: 10.1371/journal.pbio.1002528 (PMC4975452; doi:10.1371/journal.pbio.1002528)

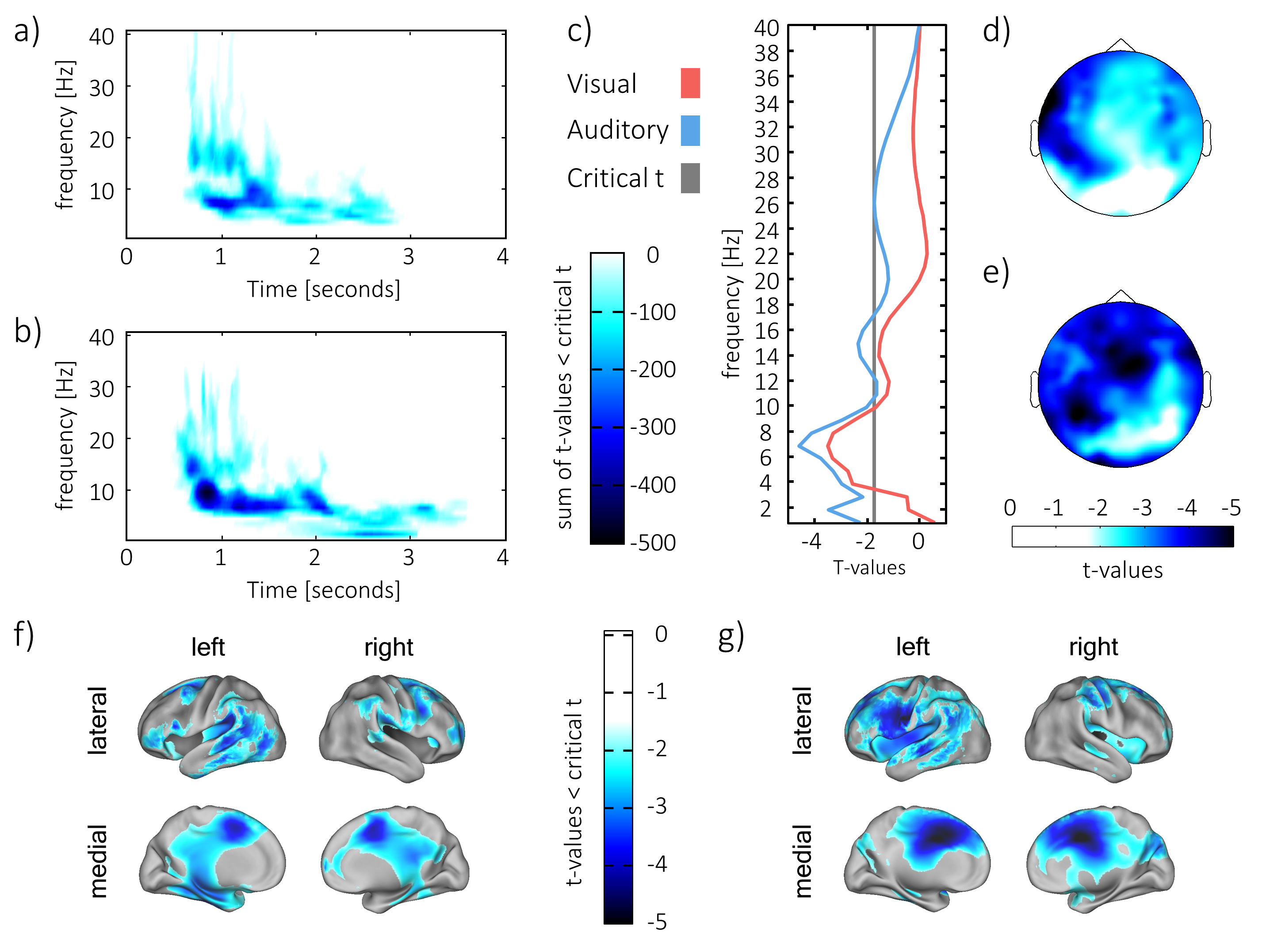

Supplement: S1 Fig — Successful memory reinstatement was associated with a cluster of broad power decreases in the lower frequencies (<30 Hz). (a-b) Sum of t-values across the electrodes in the cluster of significant differences for the visual condition (a) and for the auditory condition (b). (c) T-statistic of power decrease, averaged over electrodes and time. (d,e) Topography of power decreases in the visual condition (d) and in the auditory condition (e). Power decreases are plotted as t-values of average difference at 8 Hz between 0 and 4 s during retrieval. (f,g) Reconstruction of 8 Hz power difference in source space using an “lcmv” beamforming-algorithm in the visual (f) and in the auditory condition (g). (TIF) [file pbio.1002528.s001.tif]

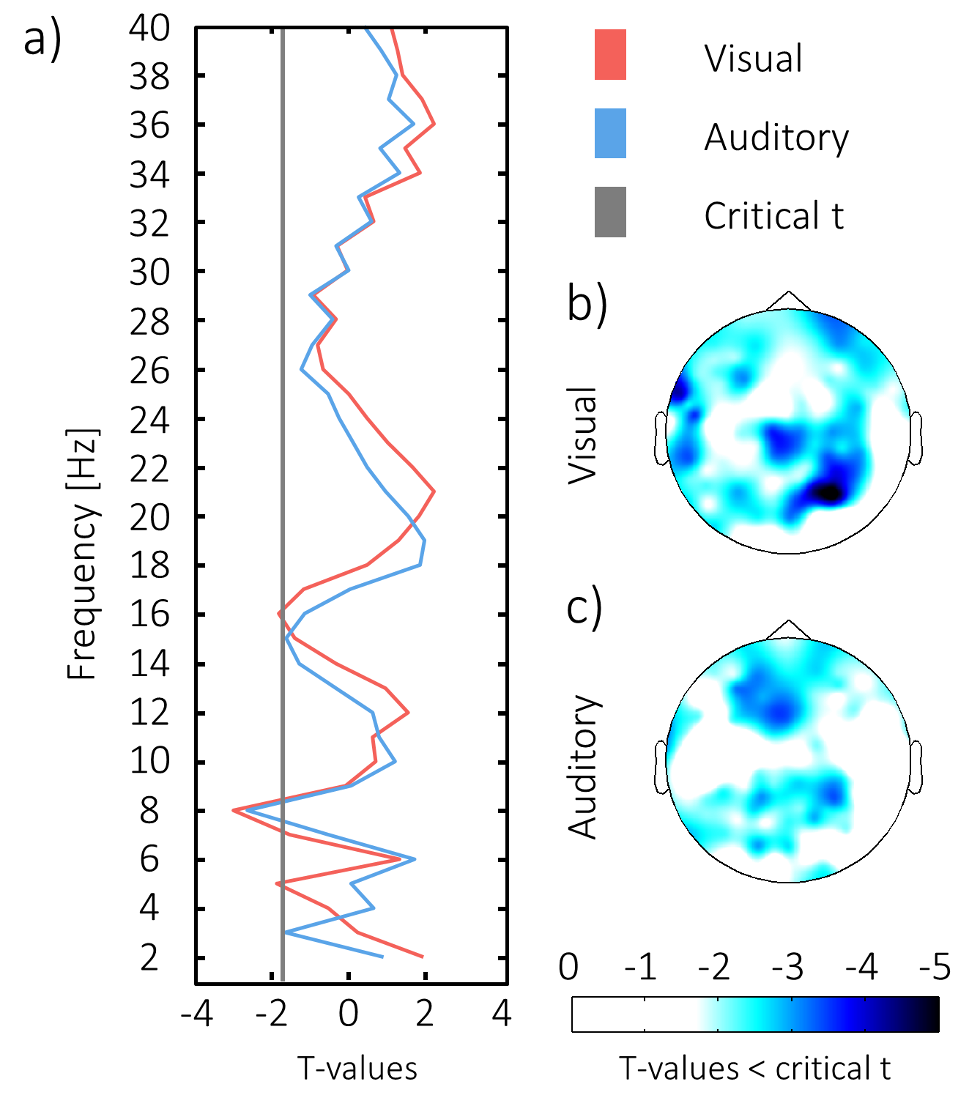

Supplement: S2 Fig — Successful memory reinstatement was associated with decreases in the stationarity of the signal. In the contrast of hits and correct rejections, the decrease peaked at 8 Hz (a). Topographies of differences in 8 Hz stationarity are shown on the right in the visual (b) and in the auditory condition (c). (TIF) [file pbio.1002528.s002.tif]

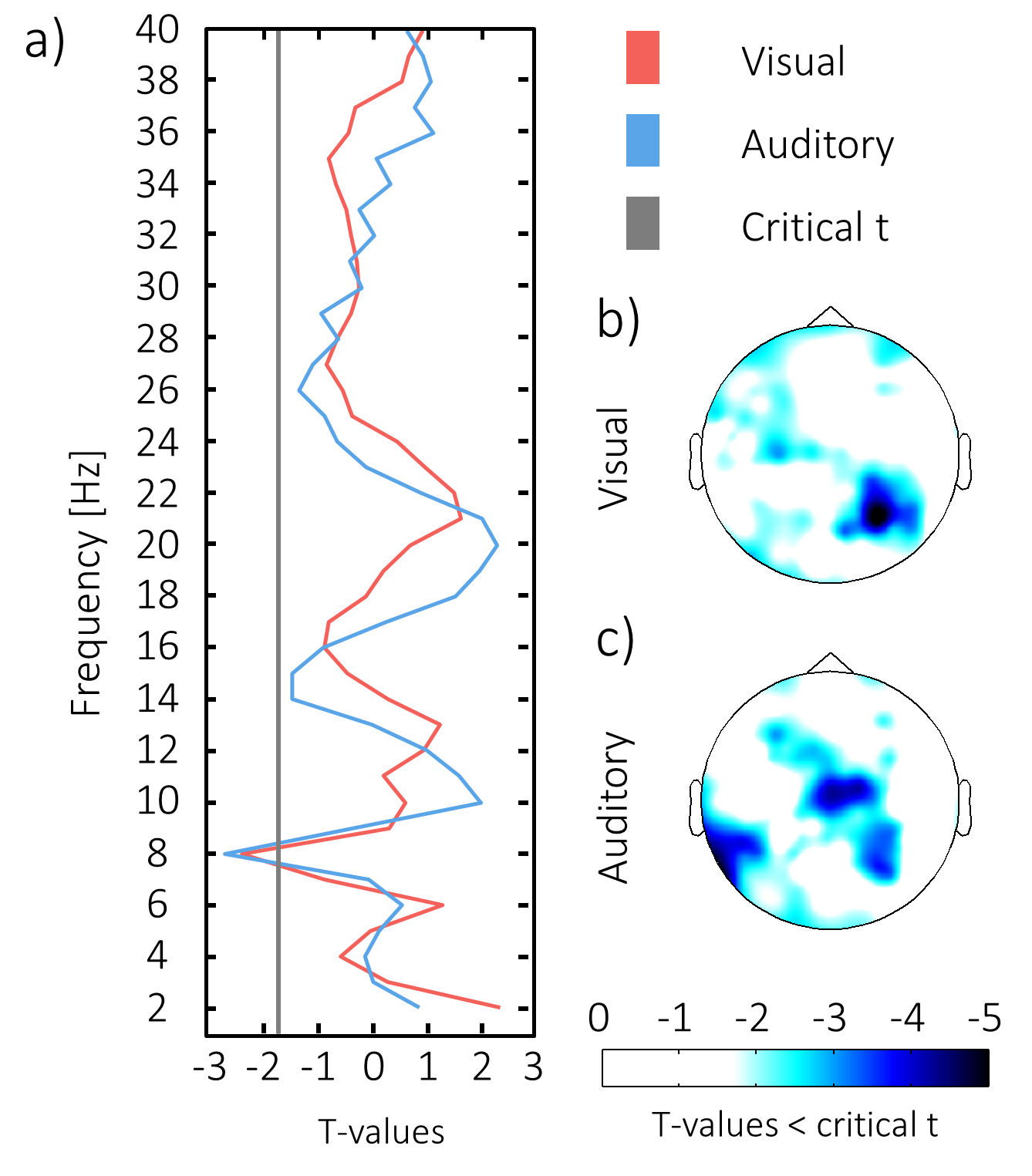

Supplement: S3 Fig — Successful memory reinstatement was associated with decreases in the stationarity of the signal. In the contrast of hits and misses, the decrease peaked at 8 Hz (a). Topographies of differences in 8 Hz stationarity are shown on the right in the visual (b) and in the auditory condition (c). (TIF) [file pbio.1002528.s003.tif]

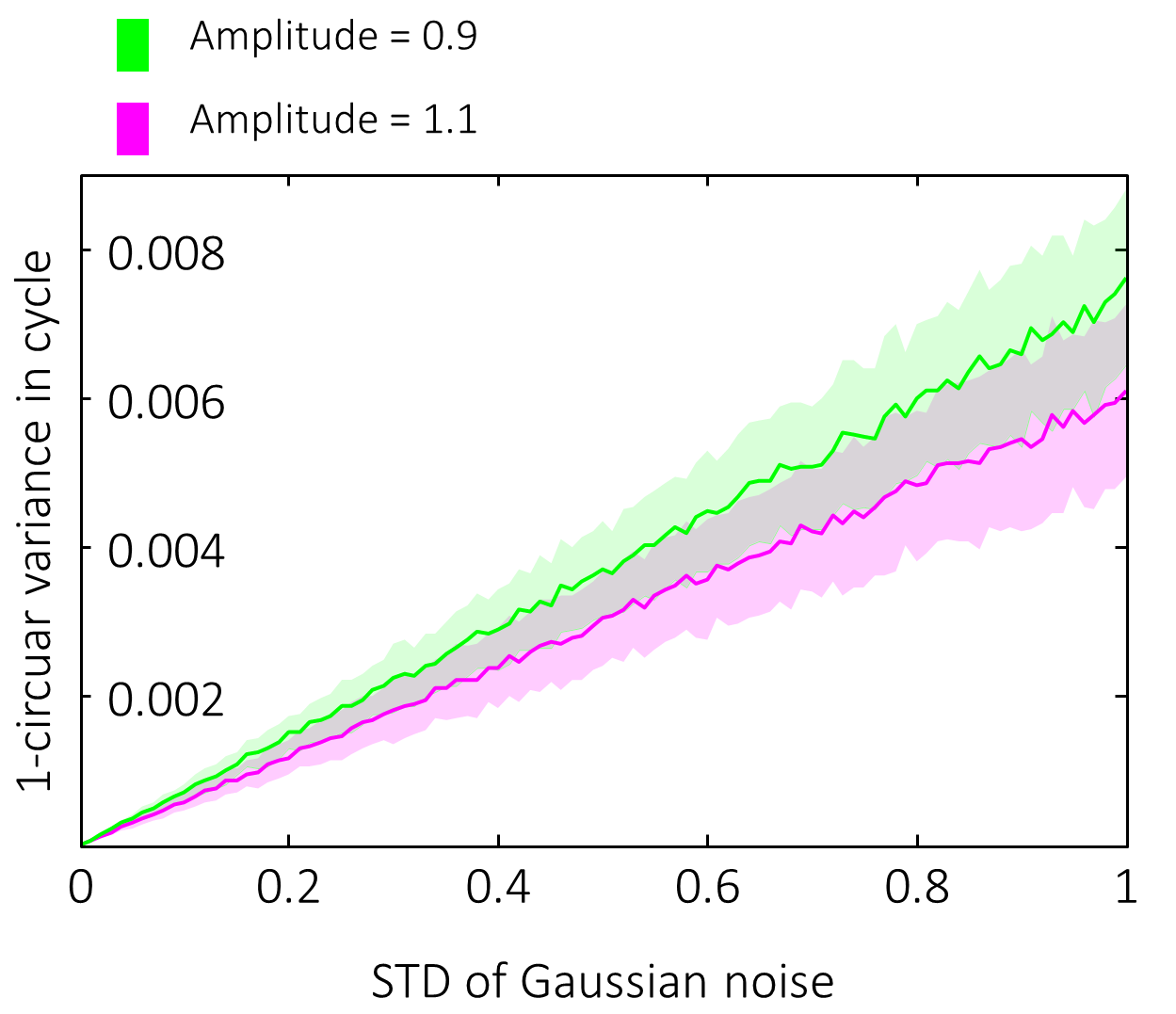

Supplement: S4 Fig — The complexity of the signal with lower amplitude is more strongly affected by noise. (TIF) [file pbio.1002528.s004.tif]
